# Ecological drivers of carrion beetle (Staphylinidae: Silphinae) diversity on small to large mammals

**DOI:** 10.1002/ece3.70203

**Published:** 2024-09-01

**Authors:** Gwen Büchner, Torsten Hothorn, Heike Feldhaar, Christian von Hoermann, Tomáš Lackner, Janine Rietz, Jens Schlüter, Oliver Mitesser, M. Eric Benbow, Marco Heurich, Jörg Müller

**Affiliations:** ^1^ Population Ecology, Animal Ecology I, Bayreuther Center of Ecology and Environmental Research (BayCEER) Faculty of Biology, Chemistry and Earth Sciences University of Bayreuth Bayreuth Germany; ^2^ Department of Biostatistics, Epidemiology, Biostatistics and Prevention Institute University of Zurich Zurich Switzerland; ^3^ Field Station Fabrikschleichach Julius‐Maximilians‐University Wuerzburg Rauhenebrach Germany; ^4^ Conservation and Research Bavarian Forest National Park Grafenau Germany; ^5^ Department of Environmental Systems Science ETH Zürich Zurich Switzerland; ^6^ National Park Monitoring and Animal Management Bavarian Forest National Park Grafenau Germany; ^7^ Department of Entomology, Department of Osteopathic Medical Specialties, College of Agriculture and Natural Resources Michigan State University East Lansing Michigan USA; ^8^ Wildlife Ecology and Conservation Biology, Faculty of Environment and Natural Resources Albert‐Ludwigs‐University Freiburg Freiburg Germany; ^9^ Institute for Forest and Wildlife Management, Inland Norway University of Applied Sciences Evenstads Vei 80, 2480 Koppang, NO‐34 Norway

**Keywords:** carrion body mass, carrion decomposition, more individuals hypothesis, transformation models

## Abstract

Silphinae (Staphylinidae; carrion beetles) are important contributors to the efficient decomposition and recycling of carrion necromass. Their community composition is important for the provision of this ecosystem function and can be affected by abiotic and biotic factors. However, investigations are lacking on the effects of carrion characteristics on Silphinae diversity. Carrion body mass may affect Silphinae diversity following the *more individuals hypothesis* (MIH). The MIH predicts a higher number of species at larger carrion because higher numbers of individuals can be supported on the resource patch. Additionally, biotic factors like carrion species identity or decomposition stage, and the abiotic factors elevation, season and temperature could affect Silphinae diversity. To test the hypotheses, we collected Silphinae throughout the decomposition of 100 carcasses representing 10 mammal species ranging from 0.04 to 124 kg. Experimental carcasses were exposed in a mountain forest landscape in Germany during spring and summer of 2021. We analysed Silphinae diversity using recently developed transformation models that considered the difficult data distribution we obtained. We found no consistent effect of carrion body mass on Silphinae species richness and, therefore, rejected the MIH. Carrion decomposition stage, in contrast, strongly influenced Silphinae diversity. Abundance and species richness increased with the decomposition process. Silphinae abundance increased with temperature and decreased with elevation. Furthermore, Silphinae abundance was lower in summer compared to spring, likely due to increased co‐occurrence and competition with dipteran larvae in summer. Neither carrion species identity nor any abiotic factor affected Silphinae species richness following a pattern consistent throughout the seasons. Our approach combining a broad study design with an improved method for data analysis, transformation models, revealed new insights into mechanisms driving carrion beetle diversity during carrion decomposition. Overall, our study illustrates the complexity and multifactorial nature of biotic and abiotic factors affecting diversity.

## Graphical abstract

To investigate the drivers of Silphinae (carrion beetle) diversity, we experimentally exposed 100 carcasses of 10 mammal species ranging from 0.4 to 124 kg. We tested the influence of the biotic factors carrion body mass (with the more individuals hypothesis (MIH) as underlying mechanism), carrion species identity and decomposition stage, and the abiotic factors temperature, season and elevation on Silphinae diversity. While the carrion body mass did not affect the Silphinae species richness (leading to rejection of the MIH), the carrion decomposition process proved to be the most important driver of Silphinae diversity; thus, our study contributes to the understanding of carrion beetle diversity.

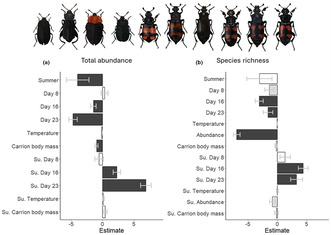

## 1 INTRODUCTION

Silphinae (Staphylinidae) are one of the few beetle subfamilies where many species are closely associated with carrion (Merritt & De Jong, 2015). They often arrive on carrion after pioneer species, such as members of the Calliphoridae family (order: Diptera; Dekeirsschieter et al., 2011; Prado e Castro et al., 2012). As invertebrate scavengers, Silphinae provide important ecosystem functions, as they promote the breakdown and recycling of organic matter (Dekeirsschieter et al., 2011; Hastir & Gaspar, 2001; Jakubec & Růžička, 2015; Kalinová et al., 2009; Ratcliffe, 1996; Von Hoermann et al., 2018; Wolf & Gibbs, 2004). Efficient carrion decomposition is vital for ecosystem nutrient and energy cycling (Moore et al., 2004; Payne, 1965; Swift et al., 1979).

The composition of scavenger communities is also important for provisioning this ecosystem function (e.g. Farwig et al., 2014; Olson et al., 2012), and can be influenced by a multitude of abiotic (e.g. Chen et al., 2009; De Jong & Chadwick, 1999; Selva et al., 2005) and biotic (e.g. Anderson, 1982; Benbow et al., 2013) factors.

In terms of abiotic factors, season (e.g. Selva et al., 2005; Voss et al., 2009), elevation (Baz et al., 2007; De Jong & Chadwick, 1999) and temperature (e.g. Chen et al., 2009; Martin‐Piera & Lobo, 1993) have been documented to affect scavenger communities. Arthropod scavenger assemblages are known to differ between the seasons (Kočárek, 2001; Růžička, 1994; Scott, 1998), with more arthropod scavenger activity reported during warmer seasons (De Jong & Chadwick, 1999; DeVault et al., 2004). Arthropod scavenger species richness (Baz et al., 2007; Farwig et al., 2014) and abundance (Farwig et al., 2014) usually decrease with increasing elevation; however, their abundance often increases with temperature (Baz et al., 2007; Chen et al., 2009; De Jong & Chadwick, 1999; Farwig et al., 2014; Martin‐Piera & Lobo, 1993; Von Hoermann et al., 2018).

The characteristics of the carrion necromass (biotic factors) such as carrion decomposition stage, carrion species identity or carrion body mass can additionally influence scavenger communities (e.g. Benbow et al., 2013; Moleón et al., 2015; Stiegler et al., 2020), including Silphinae. In contrast to decomposer communities found at other necromass such as dung (Frank et al., 2017) or deadwood (Müller et al., 2020), the influence of carrion necromass characteristics on decomposer communities is less understood.

Carrion insects such as dipterans and coleopterans are associated with certain stages of carrion decomposition (Benbow et al., 2013). The resulting insect succession throughout carrion decomposition has been used in forensic examinations to determine the postmortem interval (Lefebvre & Gaudry, 2009). Scavenging insect community composition, therefore, changes considerably throughout carrion decomposition (Benbow et al., 2013), affecting both the abundance and species diversity of the necrophagous community. The two distinct tribes of Silphinae, the Nicrophorini and the Silphini, differ in their preference for the carrion decomposition stage. While Silphini [and members of the genus *Nicrophorus* who visit larger carrion for feeding (Chauvet et al., 2008; Peck, 1986; Von Hoermann et al., 2016)] arrive at carrion during mid‐stage decay (Anderson, 1982; Matuszewski & Mądra‐Bielewicz, 2021; Payne, 1965; Prado e Castro et al., 2013), breeding Nicrophorini arrive during earlier stages of decomposition (De Jong & Chadwick, 1999; Hoback et al., 2004).

Additionally, the two Silphinae tribes differ in their preference for carrion size. In northwestern Europe, all members of the Nicrophorini belong to the genus *Nicrophorus* (Dekeirsschieter et al., 2011), which is known to prefer small carcasses (<300 g, for breeding *Nicrophorus* species; Dekeirsschieter et al., 2011; Pukowski, 1933; Scott, 1998). Therefore, in our study, the carrion size preference of the tribe Nicrophorini is determined by the carrion size preference of the genus *Nicrophorus*. The breeding *Nicrophorus* species, also known as burying beetles, bury birds, small rodents, snakes and lizards and raise their larvae in them (Anderson, 1982; Kočárek, 2003; Milne & Milne, 1976; Pukowski, 1933). However, feeding *Nicrophorus* can visit larger carrion (Chauvet et al., 2008; Peck, 1986; Von Hoermann et al., 2016). Members of the tribe Silphini tend to prefer large carrion species such as wild boar (Anderson, 1982; Anton et al., 2011; De Jong & Chadwick, 1999; Matuszewski & Mądra‐Bielewicz, 2021; Peck, 1990).

Carrion necromass constitutes a high‐quality nutrient resource pulse with low C/N ratio (Barton et al., 2013), where the carrion body mass defines the local resource size. In general, a larger local resource, in this context a larger carcass, can harbour a larger number of insects (Müller et al., 1990; Nagano & Suzuki, 2007). With a larger number of individuals, insect assemblages on larger carcasses should secondarily comprise a larger number of species, according to the m*ore individuals hypothesis* (MIH; terminology first introduced by Srivastava & Lawton, 1998). The MIH predicts the relationship between resource size (here carrion body mass) and diversity and is derived from the species‐energy theory (a more general biogeographic extension of species‐area theory; Wright, 1983). The hypothesis implies that with available chemical energy (Gibbs free energy, in our study represented by carrion necromass) abundance increases and, secondarily, diversity (Clarke & Gaston, 2006; Schuler et al., 2015; Srivastava & Lawton, 1998). A higher scavenger abundance (Stiegler et al., 2020) and species richness (Moleón et al., 2015) have been detected at carrion with higher body mass (Stiegler et al., 2020). However, these studies concentrated exclusively on vertebrate scavengers, and currently little is known on how carrion body mass drives invertebrate scavenger communities.

For carrion studies concerning scavenging insects like Silphinae, most studies have only used either a single carrion species (e.g. Payne, 1965; Von Hoermann et al., 2018: *Sus scrofa* piglets; Farwig et al., 2014; Wolf & Gibbs, 2004: *Mus musculus*) or a very limited set of species (Von Hoermann et al., 2021: *Capreolus capreolus*, *Cervus elaphus* and *Vulpes vulpes*) to test for carrion characteristics on diversity. Investigations comparing insect communities among multiple carrion species and over body mass ranges are lacking. As a result, and in contrast to litter, dung or deadwood, the ecological mechanisms driving the diversity of insects associated with carrion are not well understood (Benbow et al., 2019).

To address this lack of knowledge in carrion ecology, we experimentally exposed 100 carcasses originating from 10 mammal species representing a broad range of body masses, from 0.04 kg (stoat) to 124 kg (red deer), in a temperate mountain forest during spring and summer. We recorded Silphinae diversity (in this study represented by Silphinae abundance and species richness) throughout the carrion decomposition process. Subsequently, we employed transformation models that considered carrion species identity, carrion body mass, time since carrion exposition, on‐site air temperature, elevation above sea level (a.s.l.) and season to identify biotic and abiotic factors driving Silphinae diversity on carrion.

We hypothesized that carrion body mass, species identity and decomposition stage would affect Silphinae diversity. We expected Silphinae abundance and species richness to increase with carrion body mass due to larger resource availability. Since body mass differs among carrion species, this pattern would be reflected in species identity. Moreover, we hypothesized Silphinae abundance and species richness would change throughout carrion decomposition, with both being highest during mid‐stage decay, as breeding Nicrophorini that arrive early will still be found and the abundance and species richness of feeding Nicrophorini and Silphini, in general, will increase towards mid‐stage decay. We also expected abiotic factors to impact Silphinae abundance and species richness since previous studies have shown a positive correlation between temperature and arthropod abundance. In accordance with the lower temperatures of higher elevations or colder seasons earlier in the year, we expected lower Silphinae abundance at high elevations and during spring.

## 2 MATERIALS AND METHODS

### 2.1 Study area

**FIGURE 1:**
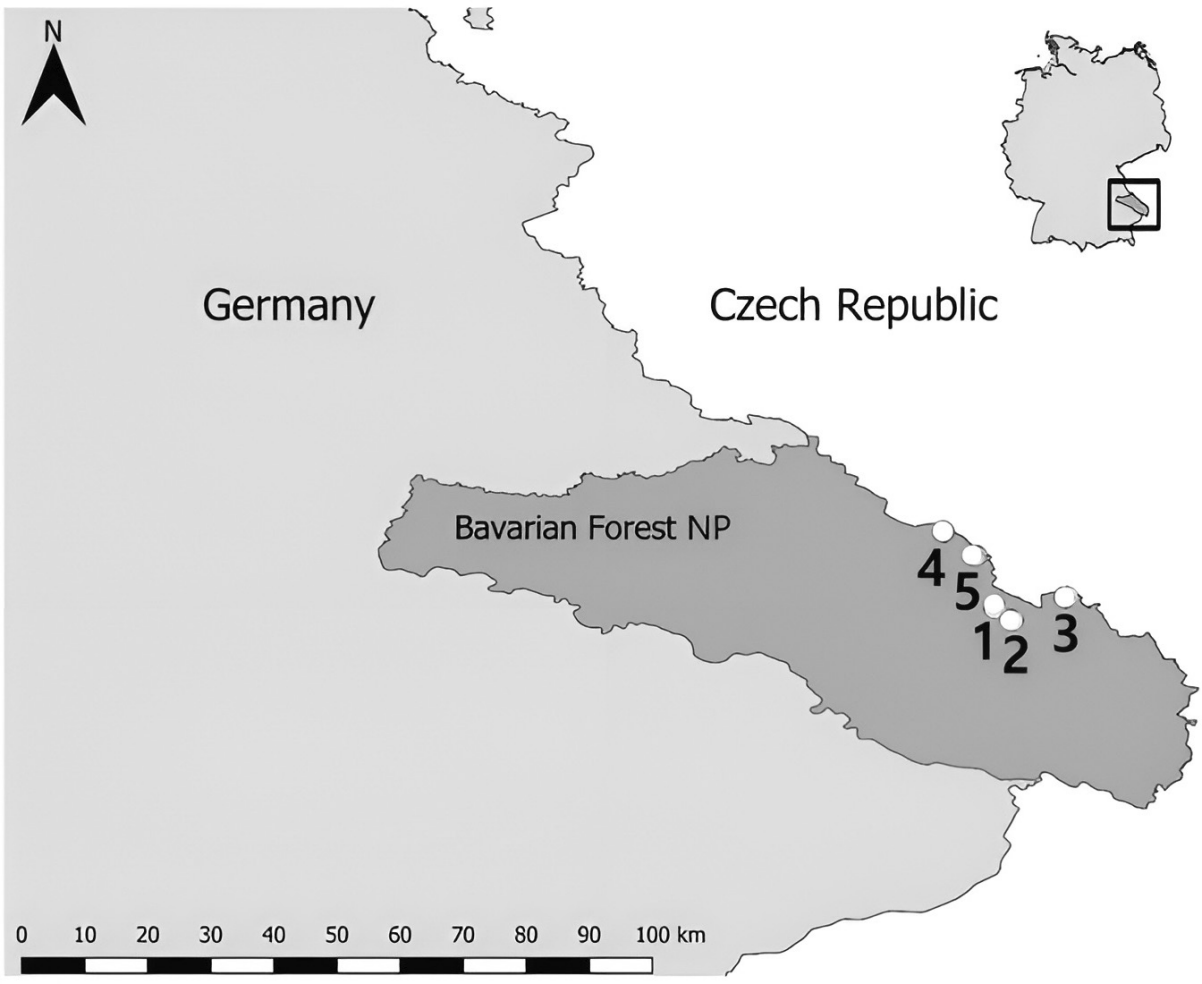
Map of Bavarian Forest National Park (NP; shape file from OpenStreetMap contributors, 2017) and surrounding area (shape file for Germany from Hijmans, 2015) with the positions of the sites 1–5 indicated by numbered marks. The map was created in QGIS (QGIS.org, 2024).

The study was conducted at five sites in the temperate montane zone (700–1300 m a.s.l., Appendix 1) of the Bavarian Forest National Park in south‐eastern Germany (Figure 1). All sites were situated in early succession forests with low canopy cover. Surrounding forests were characterized by mixed mountain forests of broadleaves and conifers. For more details on forest structure, vegetation history and management strategy, please see van der Knaap et al. (2020) and citations therein.

### 2.2 Experimental design

We provided carrion of 10 mammalian species to obtain a wide body mass range (see Table 1). One set of 10 carcasses, comprising one of each carrion species, was exposed per site once in spring (April–June; start of carrion exposure in sites 3–5 delayed due to snow) and once in summer (July) of 2021. There were five sites in total (Figure 1). The summer deployment was carried out as repeated baiting, using the same sites for multiple carcasses. New carrion was placed about 5 m next to the remains of the same carrion species during the spring deployment. At each site, the carcass set was exposed in randomized order along linear transects at the same elevation along the isohypse with a minimum intercarcass distance of 100 m to facilitate independence of replicates and reduce potential cross‐contamination among carcasses (Perez et al., 2016). A minimum distance of 80 m was kept preventing disturbance by humans. To protect carrion from being carried away by vertebrates, the Achilles tendon was secured to a wooden post with jute cord. Complete carrion removal by vertebrate or invertebrate scavengers was recorded (see Appendix 2; did not occur frequently, but mainly with smaller carrion) and no further sampling was carried out at affected locations.

**TABLE 1:** Individual body weights of carrion in kilogrammes with site number and season.

Scientific name (common name)	Site 1 body weight [kg] spring/summer	Site 2 body weight [kg] spring/summer	Site 3 body weight [kg] spring/summer	Site 4 body weight [kg] spring/summer	Site 5 body weight [kg] spring/summer
Small					
*Mustela erminea/nivalis* (stoat)	0.4/0.22	0.06/0.21	0.16/0.05	0.14/0.05	0.15/0.04
*Rattus norvegicus* (rat)	0.2/0.21	0.2/0.21	0.19/0.21	0.17/0.2	0.21/0.21
*Martes martes/foin*a (marten)	2.15/1.45	1.5/1.25	1.85/1.15	1.95/1.2	1.7/1.8
Medium					
*Procyon lotor* (raccoon)	5.95/5.55	6.2/5.7	5.75/5.25	4.75/6.1	4.65/4.8
*Vulpes vulpes* (red fox)	7.65/6.4	7.3/7	6.35/7.25	5.35/4.25	6.8/6.15
*Meles meles* (badger)	6.1/9.85	9.8/8.6	14.3/8.3	8/6.9	11.7/7.2
*Castor fiber* (beaver)	8.55/15.5	26.15/26.6	13.55/8.5	16.25/8.65	10.35/19.3
*Capreolus capreolus* (roe deer)	28.1/19.7	27.5/28.7	19.5/20.9	14.8/11.65	26.45/28.4
Large					
*Sus scrofa* (wild boar)	31.5/48	109.6/50	48/50.5	56.8/46	76/9.5
*Cervus elaphus* (red deer)	109/78.8	105.1/95	53.6/54.4	96.8/61.3	123.6/74

*Note*: The scientific and common name of the carrion species are given. The carrion species are divided into the body mass ranges small (0.04–2.50 kg), medium (>2.50–30.0 kg) and large (>30.0–125 kg).

### 2.3 Silphinae sampling

**FIGURE 2:**
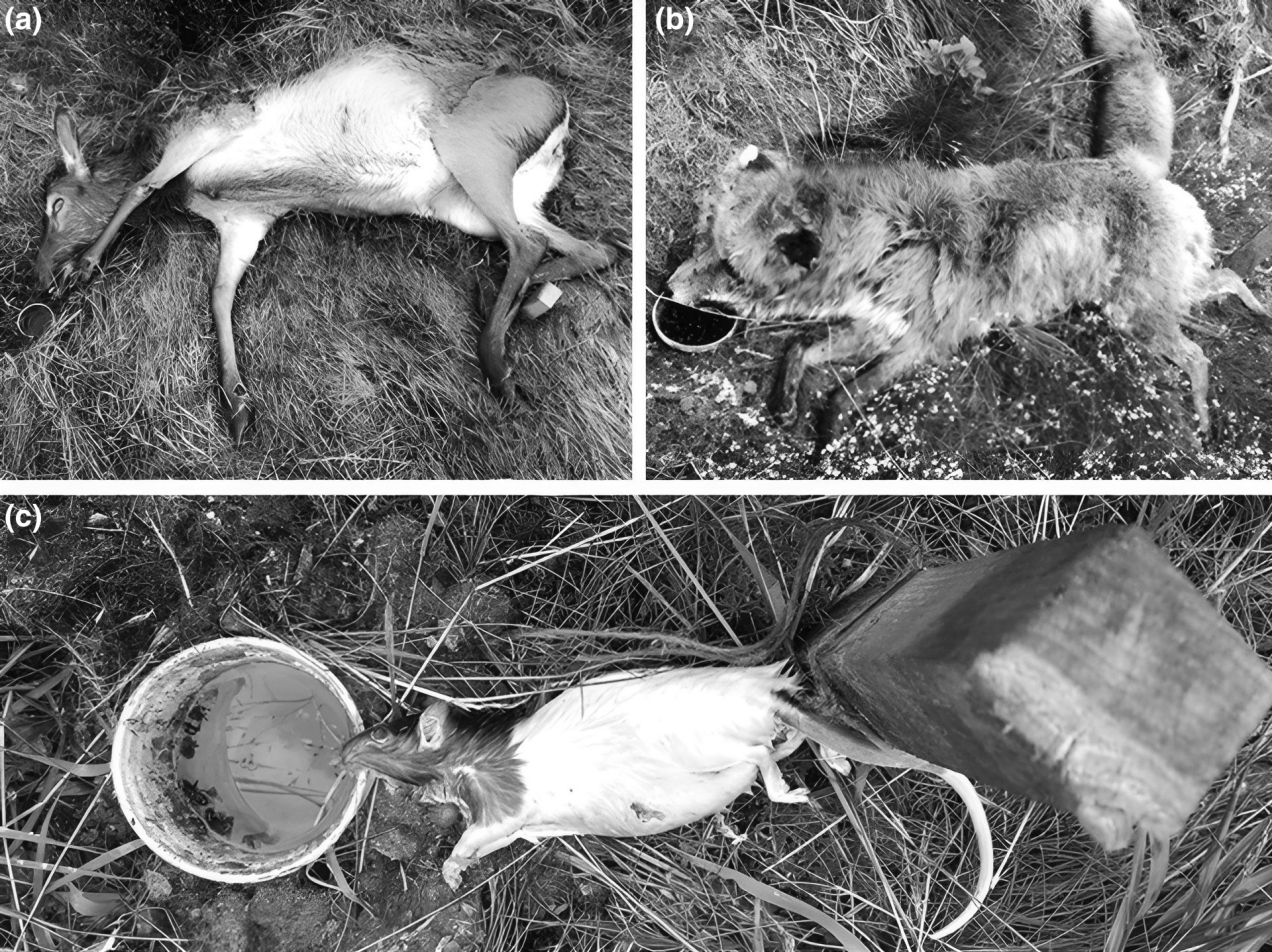
Barber pitfall traps positioned at the mouth‐opening of the carrion exemplarily shown for (a) large (*Cervus elaphus*; 53.6–123.6 kg), (b) medium (*Vulpes vulpes*; 4.25–7.65 kg) and (c) small (*Rattus norvegicus*; 170–212 g) carrion.

For Silphinae sampling, a total of four collection events were conducted on each carcass. We used Barber pitfall traps (500‐mL plastic cups filled with water mixed with a drop of unscented dish washing soap), positioned at the carcass mouth‐opening (see Figure 2), an important first colonization site for insect scavengers (e.g. Dekeirsschieter et al., 2011). The samplings took place 48 h each and were conducted in predetermined time intervals. The Barber pitfall traps were opened to start Silphinae collection on days 2, 6, 14 and 21 after carcass deployment, and emptied after 48 h on days 4, 8, 16 and 23 respectively. The trap contents were stored in 70% denatured ethanol. Silphinae specimens were separated and identified to species. Identifications of two specimens of a very rare species (*Nicrophorus sepultor*) were confirmed by an expert of the family (Jan Růžička, Prague, Czech Republic). On days 4, 8, 16 and 23, we also evaluated the decomposition stage, which we divided into the following distinguishable successive phases: fresh, putrefaction, bloated, post bloated, advanced decay and dry remains based on Goff (2009). Furthermore, mummification was included as a decomposition stage, resulting from progressive dehydration of the tissue which inhibits normal putrefactive decomposition.

To measure on‐site air temperature, we used TOMST data loggers (TMS‐4; Wild et al., 2019) placed at about 5 m from each carcass. For analyses, the mean air temperature during the 48‐h capture period was used, hereafter referred to as temperature.

### 2.4 Statistical analyses

Each carrion species was replicated five times per season (one carcass of each species per site), resulting in a total of 100 carcasses. At each carcass, four sampling events took place to be able to temporally resolve the Silphinae diversity during carrion decomposition. As a result, the Silphinae dataset consists of 400 individual abundance data points. With about 45% of zero values, the Silphinae data were heavily zero inflated (see Appendix 3), which challenges statistical modelling. We, therefore, used recently developed transformation models (Siegfried & Hothorn, 2020; Tamási & Hothorn, 2021). These models have no *a prior* assumption on data distribution, but adapt the model structure to the data by estimating a suitable transformation function. The models directly express the conditional cumulative distribution function of abundance or species richness under different experimental or environmental conditions. To test our hypotheses, we fitted transformation models for overall abundance and number of species, controlled for abundance (Gotelli & Colwell, 2001), hereafter simply referred to as species richness. We used random intercepts for carrion nested in sites to reflect the correlation structure in the observations. As fixed predictors carrion body mass, carrion species, elevation, season, temperature and day since carcass deployment were considered in the models (see Appendices 2 and 4).

**FIGURE 3:**
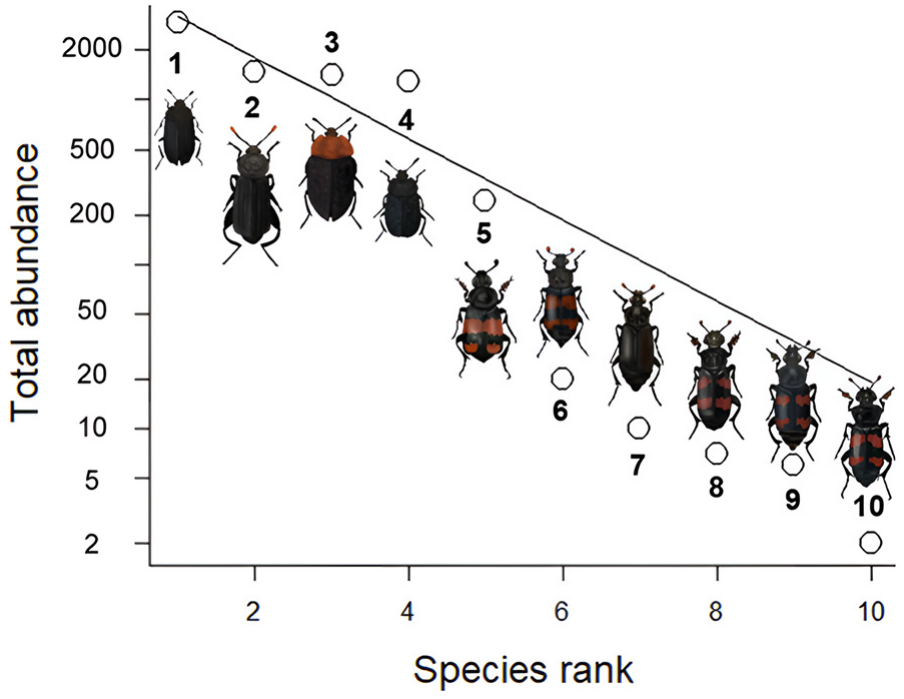
Rank abundance curve (Whittaker plot with pre‐emption fit) of Silphinae species collected at 100 carcasses of 10 different mammal species in an experimental exposure in spring and summer in this study. **1** *= Thanatophilus sinuatus*, **2** *= Necrodes littoralis*, **3** *= Oiceoptoma thoracicum*, **4** *= Thanatophilus rugosus*, **5** *= Nicrophorus vespilloides*, **6** *= Nicrophorus investigator*, **7** *= Nicrophorus humator*, **8** *= Nicrophorus interruptus*, **9** *= Nicrophorus vespillo*, **10** *= Nicrophorus sepultor*.

Regression parameters are interpretable as log‐odds ratios assumed to be constant for all possible values of the respective response variable (Siegfried & Hothorn, 2020), conditional on random intercepts (Tamási & Hothorn, 2021). Plots of model‐induced distribution functions were obtained by integrating over the estimated random effects distribution. We additionally modelled the five most abundant Silphinae species individually to gain information on species‐specific drivers. These species were *Oiceoptoma thoracicum*, *Necrodes littoralis*, *Thanatophilus sinuatus*, *Thanatophilus rugosus* and *Nicrophorus vespilloides* (Figure 3). Models M1 and M2 (see Table 2) were used for this purpose, in which the *Silphinae abundance* was replaced by the abundance of the respective species. In the models, we used the carrion species *Sus scrofa* as a reference for the species identity since *S. scrofa* is an ecologically important species that is often used in carrion studies, which increases comparability. For temporal succession, we used day 4 as a baseline (for R‐scripts, see Data Availability Statement). All analyses were conducted using R 4.2.1 (R Core Team, 2021).

**TABLE 2:** Formulas of the used models.

Model name		Model formula
M1	←	Silphinae abundance ~ season * [day + T + log_10_ (carrion body mass)] + (1|$\frac{\text{site}}{\mathrm{ID}\;\text{carrion}}$)
M2	←	Silphinae abundance ~ season * [day + T + carrion species] + (1|$\frac{\text{site}}{\mathrm{ID}\;\text{carrion}}$)
M3	←	Silphinae species richness ~ season * [day + T+ log_10_ (carrion body mass) + log_10_ (Silphinae abundance)] + (1|$\frac{\text{site}}{\mathrm{ID}\;\text{carrion}}$)
M4	←	Silphinae species richness ~ season * [day + T + log_10_ (Silphinae abundance) + carrion species] + (1|$\frac{\text{site}}{\mathrm{ID}\;\text{carrion}}$)

*Note*: Day stands for day since exposure of carrion, T refers to temperature and ID carrion stands for the individual carcasses (with unique identifier).

## 3 RESULTS

In total, we captured 7356 Silphinae individuals representing 10 species (Figure 3), from the 100 carcasses during the two deployments. With 7067 individuals, Silphini was the most prominent tribe (Appendix 5) that included the overall most abundant species, *Thanatophilus sinuatus* (2917 individuals; Figure 3). The tribe Nicrophorini was represented by 289 individuals (Appendix 5) with *Nicrophorus vespilloides* most abundant (244 individuals; Figure 3). *Nicrophorus sepultor* was detected in the Bavarian Forest National Park (BFNP) for the first time.

### 3.1 Effects of carrion characteristics on Silphinae diversity

#### 3.1.1 Carrion body mass

**FIGURE 4:**
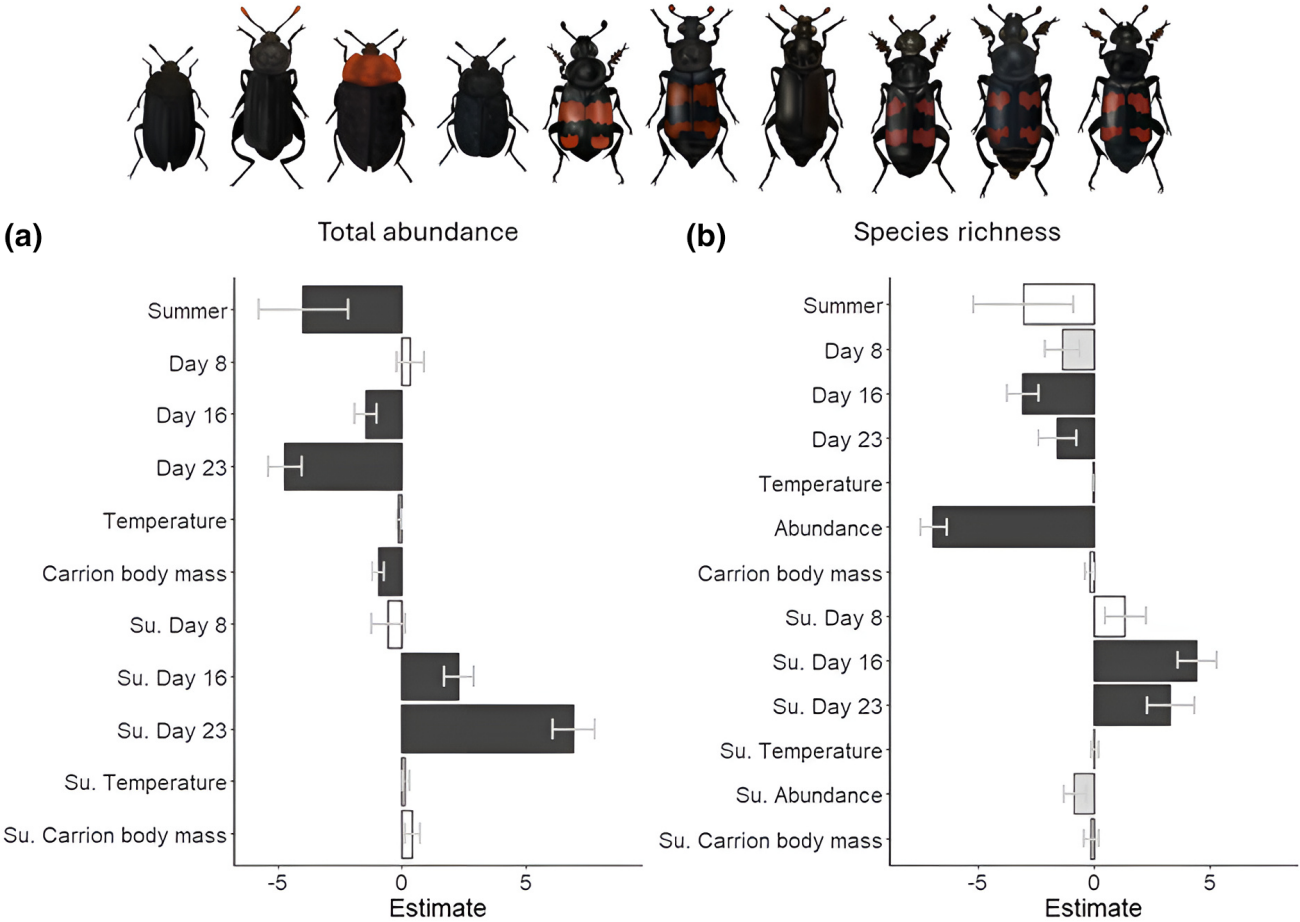
Bar plots depicting the estimates (with standard errors in) for the predictors calculated by the transformation models (reference for day since carrion exposure = day 4, su. = summer; models: M1 → abundance, M3 → species richness, see Table 2) for Silphinae total abundance and species richness. Statistical significance is indicated by colour of the bars [black bars = significant (*p* < .05), grey bars = marginally significant (.5 < *p* < .1), open bars = not significant (*p* > .1)]. Algebraic signs of the estimates are opposite to the direction of the biological effect of the predictors, that is, a negative sign means a positive biological effect.

**FIGURE 5:**
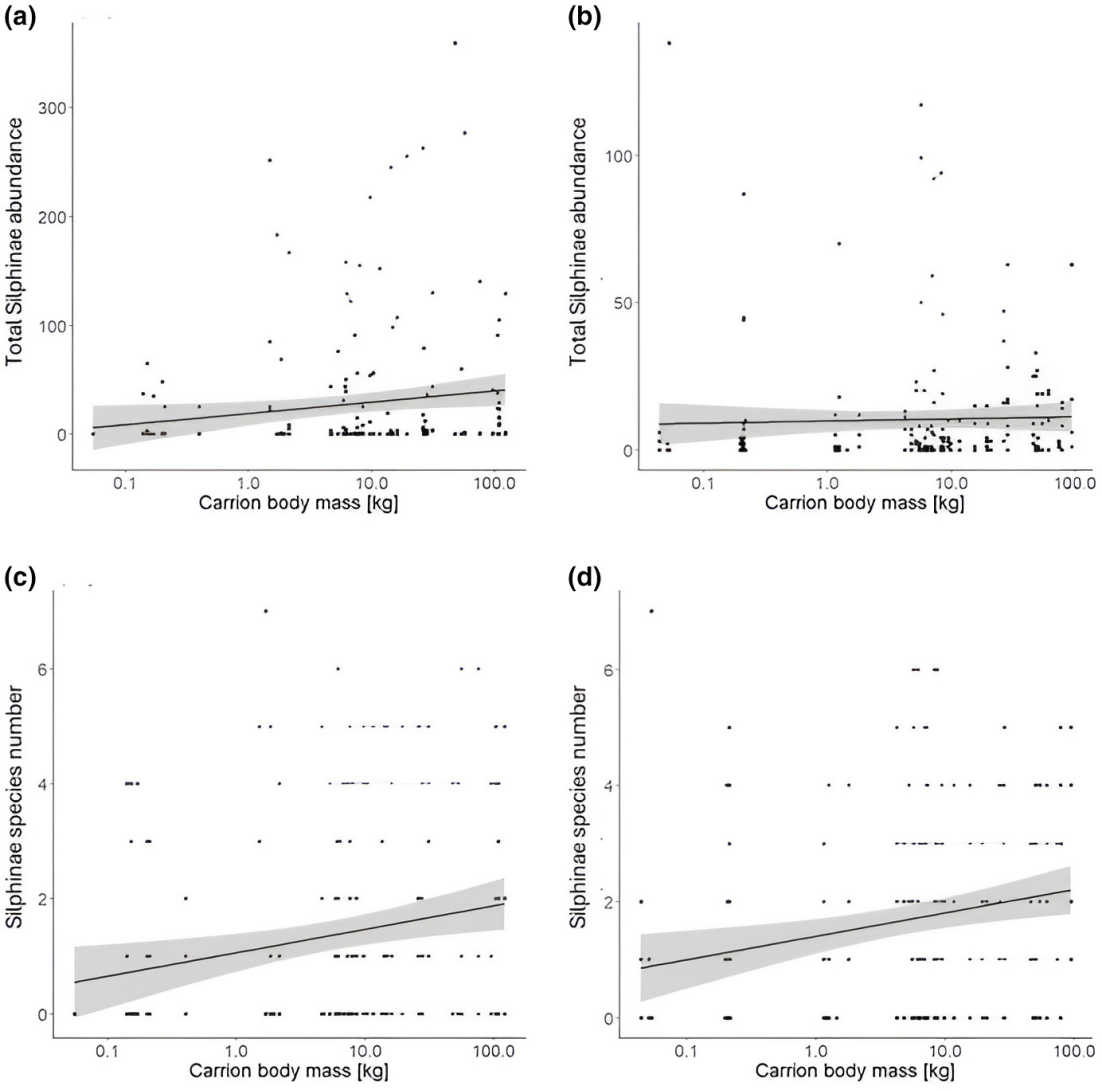
Total Silphinae abundance (a, b) and Silphinae species number (c, d) for the decadic logarithm of carrion body mass in kilogrammes shown for spring (a, c) and summer (b, d). The regression lines for the relationships between Silphinae abundance/species number and decadic logarithm of carrion body mass are given.

For both seasons, there was a positive effect of carrion body mass on Silphinae abundance (Figures 4 and 5a,b, Appendices 6 and 7). However, body mass did not affect Silphinae species richness (Figures 4 and 5c,d, Appendices 6 and 7).

**FIGURE 6:**
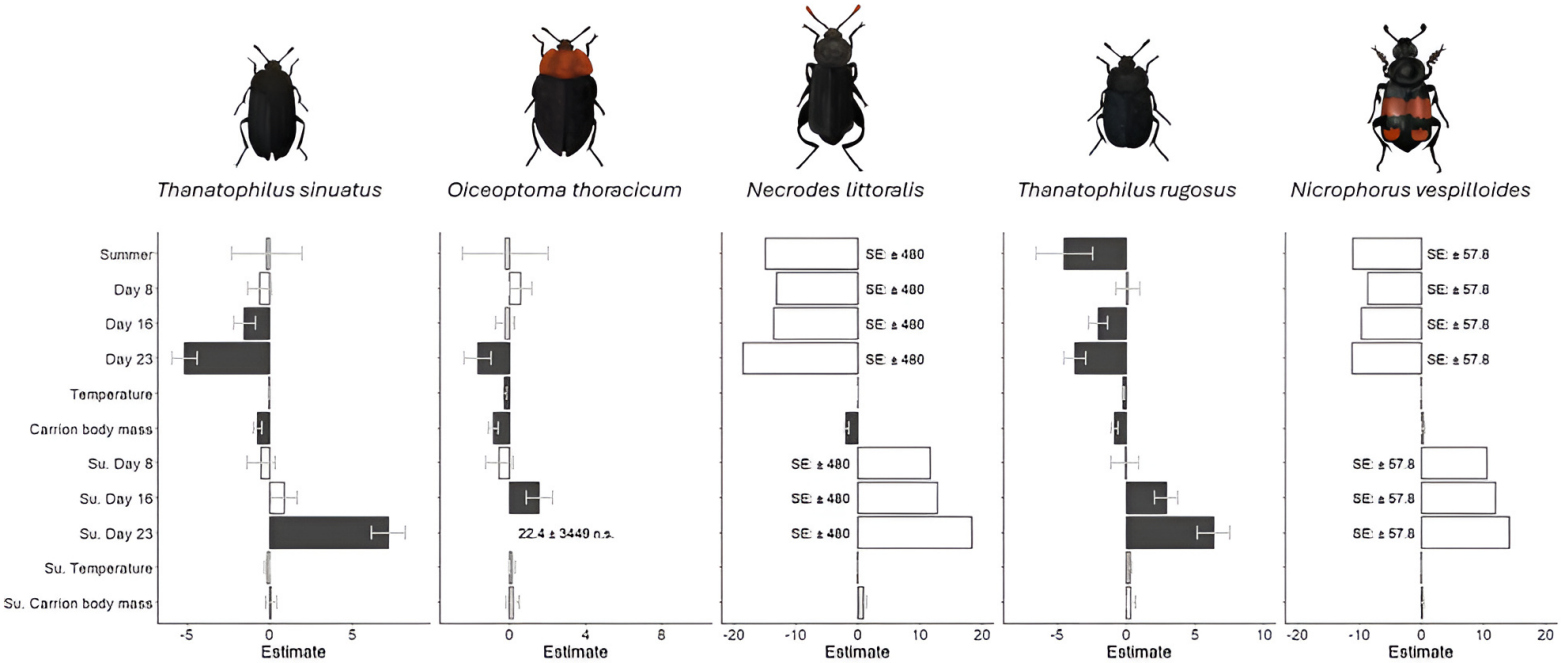
Bar plots depicting the estimates (with standard errors in) for the predictors calculated by the transformation models (reference for day since carrion exposure = day 4, su. = summer; model: M1 ➔ abundance, see Table 2) for the five most abundant Silphinae species. Statistical significance is indicated by colour of the bars [black bars = significant (*p* < .05), grey bars = marginally significant (.5 < *p* < .1), open bars = not significant (*p* > .1)]. Algebraic signs of the estimates are opposite to the direction of the biological effect of the predictors. Standard errors (SE) or estimates, that are not statistically significant (n.s.) with values so large, they would distort the presentation are given as numeric values.

Carrion body mass had a positive effect on abundance of the five most common Silphinae species (Figure 6, Appendices 6 and 7): *Thanatophilus sinuatus* (marginally significant), *Necrodes littorals*, *Oiceoptoma thoracicum* and *Thanatophilus rugosus* but no effect on *Nicrophorus vespilloides*. The effect was consistent in both seasons except for *N. littoralis*, where it was marginally significantly lower in summer compared to spring.

#### 3.1.2 Carrion species identity

There was no consistent effect of carrion species identity on abundance or species richness of Silphinae (see Appendices 8 and 9). In spring, carcasses of *Mustela erminea/nivalis*, *Rattus norvegicus*, *Procyon lotor* (marginally significant), *Vulpes vulpes* and *Capreolus capreolus* had a significant negative effect on Silphinae abundance compared to the reference species *Sus scrofa*, that is, abundance and species richness on the former carrion species was lower compared to *S. scrofa*. During summer, however, the effect was opposite, with carcasses of *M. erminea/nivalis* (marginally significant) having a positive influence on Silphinae abundance. Similarly, the negative influence of *Meles meles* carcasses (compared to *S. scrofa*) on Silphinae species richness, that was detected in spring, was opposite in summer. Furthermore, the only other effect of carrion species' identity on beetle species richness was a marginally significant negative effect of *Castor fiber* during spring (see Appendices 8 and 9).

Similar to the overall results, there were no clear effects of carrion species identity on the abundance of the five most common Silphinae species. Nevertheless, the lower abundances of *T. sinuatus*, *O. thoracicum* and *T. rugosus* detected at the *M. erminea/nivalis* and *R. norvegicus* carcasses (compared to *S. scrofa*) suggested a trend for lower Silphinae abundances at smaller carrion species (Appendices 8 and 9). However, this effect is only evident for these three Silphinae species and was inconsistent for *T. rugosus* throughout the seasons.

#### 3.1.3 Carrion decomposition stage

**FIGURE 7:**
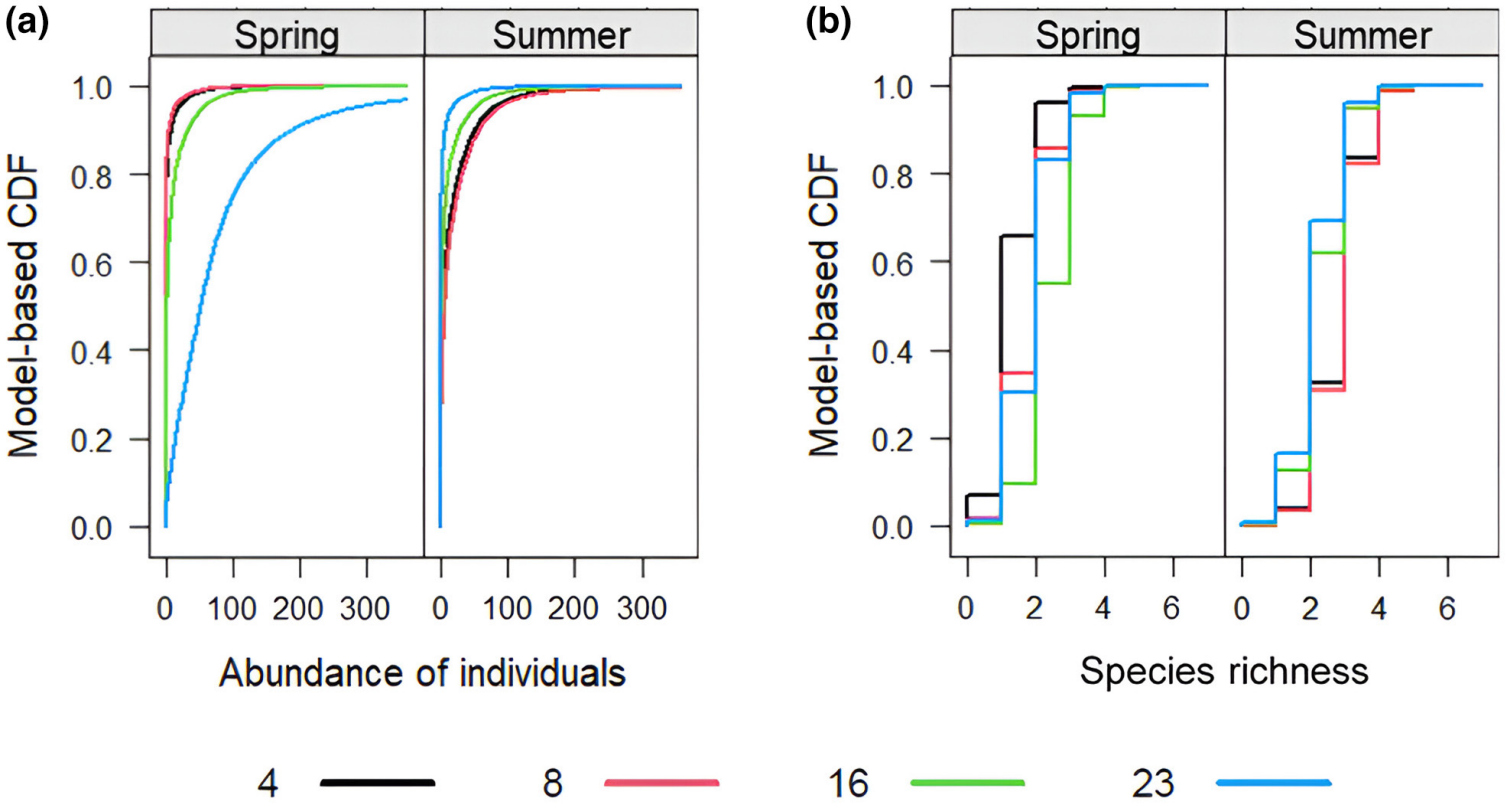
Trellis display of the model‐based cumulative distribution functions (CDFs) of (a) Silphinae abundance and (b) Silphinae species richness for the days since deployment of the carrion (indicated by the colouration of the graphs) for spring and summer. Corresponding Trellis displays for the empirical CDFs in Appendices 10 and 11.

The transformation models revealed that day since carcass deployment, and therefore, advancing carrion decomposition, influenced Silphinae abundance and species richness, with high effect strength but opposing directions in spring and summer (see Figure 4, Appendices 6 and 7). Silphinae abundance [Figure 7a: spring; note: as the trellis displays of the model‐based CDFs are very close to the empirical ones (compare Appendix 10), this indicates a good fit] and species richness (Figure 7b: spring) were significantly higher on days 16 and 23 compared to day 4 in spring. Furthermore, species richness was marginally significantly higher on day 8 compared to day 4. For Silphinae abundance, the absolute effect strength increased from day 16 to day 23 (see Figures 4 and 7, Appendices 6 and 7). Silphinae abundance (Figure 7a: summer) and species richness (Figure 7b: summer) were significantly reduced on days 16 and 23 in summer. For the abundance, the effect strength of the day since exposure increased from 16 to 23 (see Figures 4 and 7, Appendices 6 and 7).

The seasonal differences in Silphinae abundance and species richness follow the increased rate of decomposition in summer compared to spring (see Appendix 12). During spring, Silphinae abundance was highest on day 23 (Figure 7a, spring: blue line) and species richness on day 16 (Figure 7b, spring: green line), while during summer Silphinae abundance and species richness were highest on days 4 and 8 (Figure 7a,b, summer: black and red lines).

**FIGURE 8:**
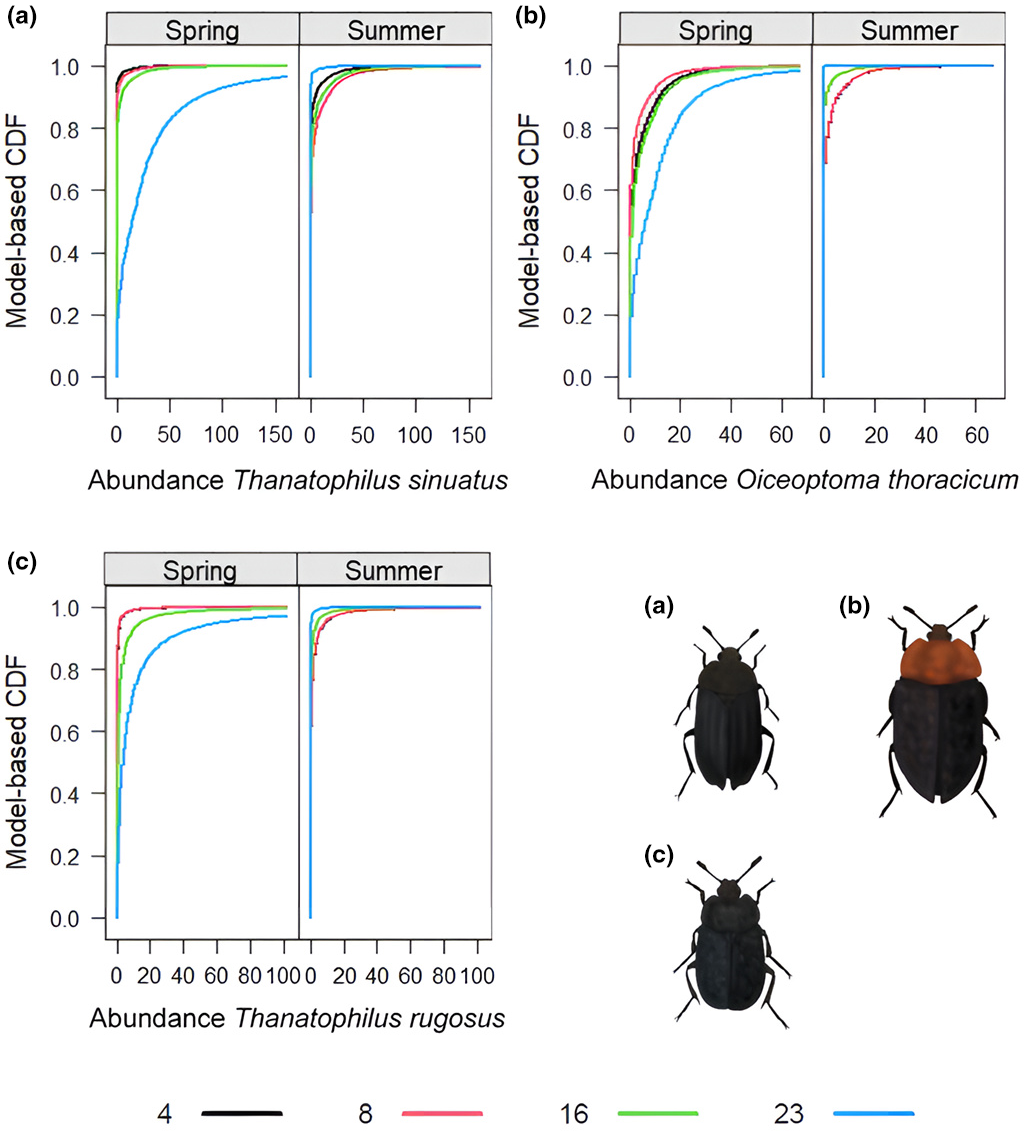
Trellis display of the model‐based CDFs (cumulative distribution functions) of the abundances of (a) *Thanatophilus sinuatus*, (b) *Oiceoptoma thoracicum* and (c) *Thanatophilus rugosus* for the days since deployment of the carrion (indicated by the colouration of the lines in the graphs) for spring and summer. Corresponding Trellis display for the empirical CDF in Appendices 17, 18 and 19.

Day since carcass deployment, and therefore advancing carrion decomposition, affected the abundances of three out of five species. While day since deployment did not affect the abundances of *N. littoralis* (Figure 6, Appendices 13 and 14) and *N. vespilloides* (Figure 6, Appendices 15 and 16), it did on *T. sinuatus* (Figure 8a), *O. thoracicum* (Figure 8b) and *T. rugosus* (Figure 8c; Appendices 6 and 7).

The abundances of *T. sinuatus* and *T. rugosus* were higher on days 16 and 23 compared to reference day 4. The absolute effect strength for both species increased from day 16 to day 23. Furthermore, the abundance of *O. thoracicum* was significantly higher on day 23 than on day 4, and *T. sinuatus* abundance was significantly lower on day 23 in summer compared to day 4 in spring. The abundance of *T. rugosus* was significantly lower on days 16 and 23 in summer compared to the reference with increasing effect strength from days 16 to 23. *O. thoracicum* showed a significantly lower abundance on day 16 in summer compared to day 4 in spring (Figure 6, Appendices 6 and 7).

**FIGURE 9:**
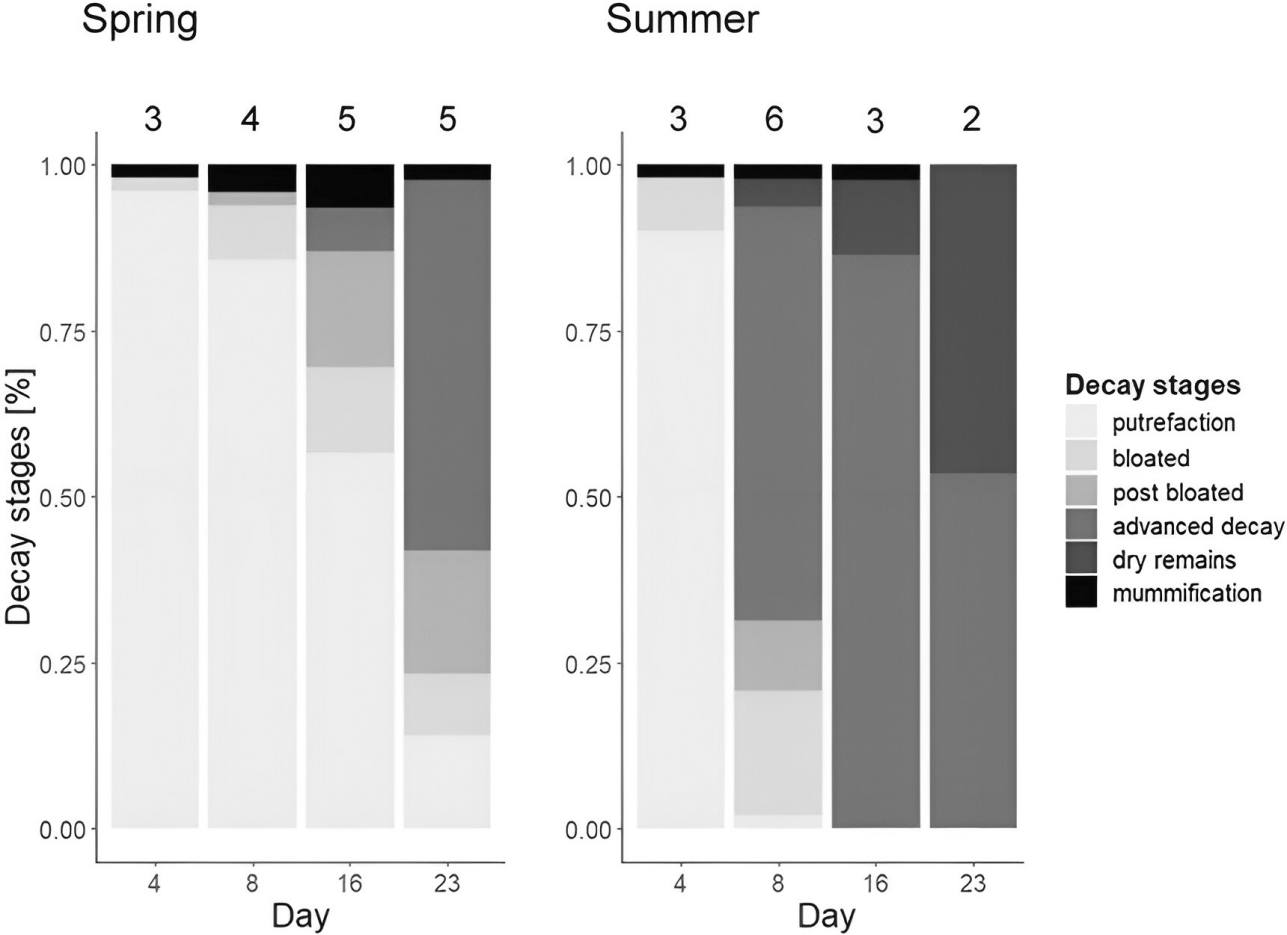
Progression of carrion decomposition over the sampling days for all 50 carcasses in each season. Greyscales of the bars depict the ratio of decomposition stages of the carcasses per day. Total number of decomposition stages per day is given above the bars. It should be noted that mummification inhibits normal putrefactive decomposition, as it is due to progressive dehydration of the tissue.

Overall, the number of different carrion decomposition stages found on the same sampling day ranged over time from 3 to 5 in spring and 2 to 6 in summer, when all carcasses are pooled by season. This number was highest on days 16 and 23 in spring (Figure 9: spring → five different decomposition stages) and day 8 in summer (Figure 9: summer → six different decomposition stages).

#### 3.1.4 Silphinae abundance

Silphinae species richness significantly increased with abundance, with the effect higher in summer than in spring (Figure 6, Appendices 6 and 7).

### 3.2 Effects of abiotic factors on Silphinae diversity

#### 3.2.1 Elevation

To test for the effect of elevation on Silphinae diversity (Appendix 20), we included elevation as a predictor in the models (model formulas in Appendix 4, graphs depicting the bar plots of the estimates with standard error for the models EM1, EM3, and EM1 modelled for the abundances of the five most abundant Silphinae species individually in Appendices 21 and 22, results for the predictors in Appendix 23). These models do not represent the simplest explanatory approach, as temperature is the most important influence of elevation and is already included in other models. Therefore, models that include elevation were used exclusively to decipher the associations with temperature. The Silphinae abundance decreased with increasing elevation (Appendices 21 and 23), but there was no effect on species richness. The individual models of the five most common Silphinae species also showed a decrease in abundance for *O. thoracicum* and *T. sinuatus*. In contrast, *T. rugosus* abundance increased with elevation (Appendices 22 and 23). For all observed effects of elevation, the effect strength was comparatively very low.

#### 3.2.2 Season

Silphinae abundance, but not species richness, was significantly higher in spring compared to summer (Figure 4, Appendices 6 and 7). When analysing the five most common Silphinae species individually, only the abundance of *T. rugosus* was significantly higher in spring (Figure 6, Appendices 6 and 7).

#### 3.2.3 Temperature

**FIGURE 10:**
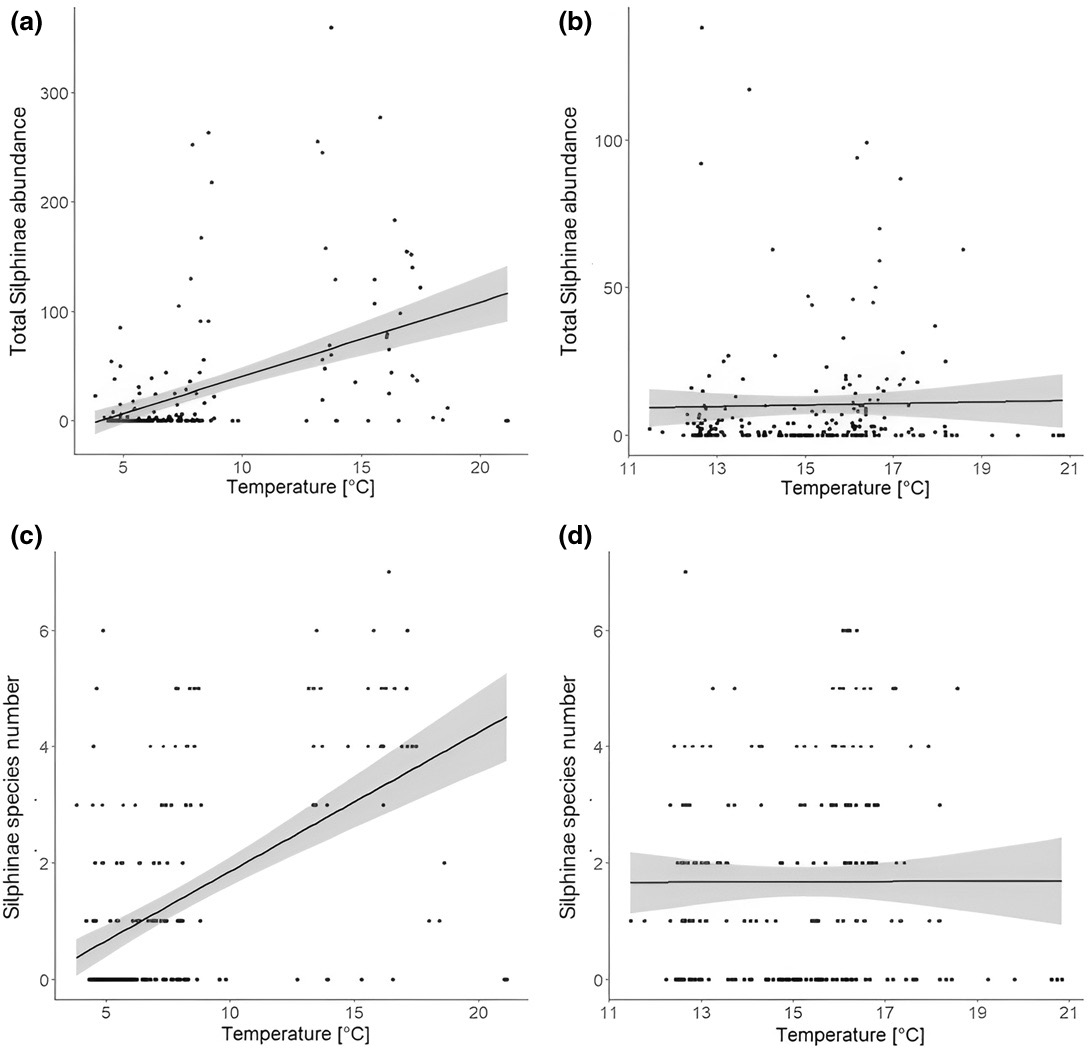
Total Silphinae abundance (a, b) and Silphinae species number (c, d) for the mean air temperature in degree Celsius shown for spring (a, c) and summer (b, d). The regression lines for the relationships between Silphinae abundance/species number and temperature are given.

Silphinae abundance, but not species richness, significantly increased with temperature (Figures 4 and 10, Appendices 6 and 7). This effect did not differ between seasons (Figure 4, Appendices 6 and 7).

Temperature influenced the abundance of two Silphinae species, with *O. thoracicum* and *T. rugosus* abundances increasing with temperature. However, this was not consistent between the seasons, since in summer temperature had no effect on the abundance of *O. thoracicum* and even negatively affected *T. rugosus* abundance (Figure 6, Appendices 6 and 7).

## 4 DISCUSSION

Our experiment with carrion originating from different species and with a broad body mass range did not generally support the *more individuals hypothesis* (MIH). The MIH was rejected since Silphinae abundance, but not richness, increased with carrion body mass (availability of chemical energy). However, the species richness, controlled for abundance, increased with the decomposition process. The changes of species richness differed between seasons, due to Silphinae associating with certain decomposition stages, and accelerated decomposition in summer compared to spring. Overall, our study illustrates the complexity and multifactorial drivers of carrion Silphinae diversity. Before discussing the ecological findings, we first evaluate the advantages and disadvantages of the methodology applied.

### 4.1 Method discussion

We used Barber pitfall traps to track Silphinae diversity throughout carrion decomposition. In contrast to comprehensive but more invasive (Melbourne, 1999) search activities on carcasses, Barber pitfall traps only capture a portion of Silphinae diversity, but they sample continuously and therefore reduce temporal sampling bias (Topping & Sunderland, 1992). However, there are discrepancies in the collection of different beetle families between pitfall trapping and active sampling (Zanetti et al., 2016). Since we investigated a single carrion beetle subfamily, this bias should be negligible. Pitfall traps are generally considered appropriate for obtaining community information (Jarošík, 1992; Von Hoermann et al., 2021, 2022, 2023) and relative abundances (Mommertz et al., 1996) of surface‐active invertebrates with distinct trophic roles (Knapp et al., 2016), such as predatory necrophilous Silphinae. Since pitfall traps have been successfully used in previous studies on carrion‐associated invertebrate diversity and community structure (e.g. Von Hoermann et al., 2020, 2021; Weithmann et al., 2021), their use in our study provides robust comparability.

### 4.2 Effects of carrion characteristics on Silphinae diversity

#### 4.2.1 Carrion body mass

Contrary to the MIH, the amount of a carrion necromass was not a significant driver of Silphinae diversity. Even though the overall total abundance and the abundances of some of the five most common Silphinae species increased with carrion body mass, the species richness was not affected. The MIH is not often supported by empirical research. Another study in the Southern Rocky Mountains that used Silphinae rejected the MIH as an explanation for diversity (McCain, 2021). Likewise, a study on dung beetles did not detect a relationship between food resource amount (dung) and beetle abundance and diversity (Gebert et al., 2020). These studies support our results of a minor role of resource amount (carrion body mass: available chemical energy) as a mechanism driving Silphinae diversity. Our findings, therefore, fit well into the discussion on the generality of the MIH hypothesis across taxa (e.g. McCain, 2021; McCain et al., 2018; Storch et al., 2018).

#### 4.2.2 Carrion species identity

Our results support other research showing Silphinae prefer larger carrion species (Anderson, 1982; Anton et al., 2011; De Jong & Chadwick, 1999; Mądra‐Bielewicz et al., 2017; Peck, 1990; Watson & Carlton, 2005), since a larger resource provides food resources that support large numbers of individuals (Anderson, 1982; Watson & Carlton, 2005). That the effect is not entirely consistent throughout the seasons could be explained by the increased co‐occurrence and competition of Diptera larvae (mainly blow flies) during summer. There have been previous reports of food resource competition for *N. littoralis* (Matuszewski & Mądra‐Bielewicz, 2021; more detailed information on this in the sub‐item season), which may be relevant for other Silphinae species.

For the two smallest carrion species (i.e. rat and stoat), we found low Silphinae abundance. In this context, it is important to mention that *Nicrophorus* species [only genus of the tribe Nicrophorini in northwestern Europe (e.g. Dobler & Müller, 2000; Sikes et al., 2002)] use small carrion (< 300 g) for reproduction (Dekeirsschieter et al., 2011; Pukowski, 1933; Scott, 1998). Classically, one beetle pair buries a recent carcass and uses it to brood larvae (Kočárek, 2003; Milne & Milne, 1976; Pukowski, 1933). A carcass claimed and buried by a beetle pair is, therefore, not accessible to others, which should lead to a low Silphini abundance found at carcasses small enough for burial (e.g. rat or stoat). Furthermore, especially during ovary maturation (Dekeirsschieter et al., 2011; Pukowski, 1933), *Nicrophorus* species can be found feeding on large carrion (Chauvet et al., 2008; Peck, 1986; Von Hoermann et al., 2016).

#### 4.2.3 Carrion decomposition stage

The progress of carrion decomposition strongly affected Silphinae abundance and species richness. Thus, carrion decomposition process (and other scavenger presence) may be a more important driver of Silphinae diversity than resource amount (carrion body mass). It was striking that Silphinae abundance and species richness were greatest on the days with the highest numbers of different carrion decomposition stages (when the decomposition stages were pooled for all carcasses, per day and season). The increase in Silphinae species richness with carrion decomposition supports the framework of Benbow et al. (2019) where it was hypothesized that two patches of carrion at different decomposition stages support greater diversity compared to the same resource patches with the same decomposition stage.

The two Silphinae tribes prefer different stages of carrion decomposition: breeding Nicrophorini are linked to earlier decomposition stages (De Jong & Chadwick, 1999; Hoback et al., 2004) compared to Silphini (Anton et al., 2011; De Jong & Chadwick, 1999) [and *Nicrophorus* species that visit larger carrion for feeding (Chauvet et al., 2008; Peck, 1986; Von Hoermann et al., 2016)] that are primarily associated with mid‐stage decay (Anderson, 1982; Matuszewski & Mądra‐Bielewicz, 2021; Payne, 1965; Prado e Castro et al., 2013). An exact assignment of the decomposition stages recorded during our study, corresponding to ‘mid‐stage decay’ was not possible, since the subdivision of carrion decomposition varies largely throughout literature (e.g. compare Payne, 1965 to Prado e Castro et al., 2013). Furthermore, it is not yet known if individual Silphinae species within the two tribes have specific preferences for carrion decomposition stages. In our study, we found temporal shifts in abundance of the four Silphini species, supporting niche differentiation at the species level; however, further research is needed.

#### 4.2.4 Silphinae abundance

We found that Silphinae species richness increased with abundance, which appears to support the MIH. However, the underlying mechanism of the MIH that more available chemical energy leads to higher abundance and then secondarily to higher diversity (Clarke & Gaston, 2006; Schuler et al., 2015; Srivastava & Lawton, 1998) is not supported. Even though Silphinae abundance increased with carrion body mass, the same was not observed for the Silphinae species richness, which contradicts the underlying mechanism.

### 4.3 Effects of biotic factors on Silphinae diversity

#### 4.3.1 Elevation

With our findings, we can confirm the frequently observed trend of decreasing abundance but not species richness of invertebrate scavengers with increasing elevation, caused by a decrease in temperature (e.g. Baz et al., 2007; De Jong & Chadwick, 1999; Farwig et al., 2014; Martin‐Piera & Lobo, 1993). As effect strengths of elevation were quite weak and the effect not entirely consistent over individual species, elevation seems to play a rather minor role as a driver of Silphinae community composition compared to other abiotic factors.

#### 4.3.2 Season

The Silphinae community composition differed between the two seasons. Differences in composition of Silphinae assemblages among seasons are known to be associated with variation in species‐specific temporal activity (Kočárek, 2001; Růžička, 1994; Scott, 1998). Such variation may be a result of temporal niche differentiation to reduce interspecific resource competition (Anderson, 1982; Hocking et al., 2007; Ohkawara et al., 1998; Peck, 1990). Seasonal compositional differences of invertebrate scavenger assemblages were observed in several previous studies (Burkepile et al., 2006; Farwig et al., 2014; Selva et al., 2005; Voss et al., 2009; Wilson & Wolkovich, 2011). During summer, Silphinae species number was higher when *N. investigator*, *N. interruptus* and *N. sepultor* exclusively occurred during this season (see Appendix 24). Previous studies, which also exclusively captured *N. investigator* and *N. interruptus* during summer, support our findings (Aleksandrowicz & Komosiński, 2005; Hastir & Gaspar, 2001; Kočárek, 2003). However, the higher abundance of *N. vespilloides* during summer is not consistent with studies showing higher abundances in spring (Dekeirsschieter et al., 2011; Kočárek, 2003). An explanation may be that our study was conducted in the temperate montane zone (700–1300 m a.s.l.), where the climatic conditions found in other study areas during spring occur here only in summer. Furthermore, a clear association of *N. vespilloides* with the spring season was not always found; Růžička (1994) reported *N. vespilloides* to be active from April to December with a weak peak from May to the middle of October.

Although the species richness was higher in summer, the vast majority of Silphinae individuals (72%) were captured in spring, including *O. thoracicum*, *T. rugosus*, *T. sinuatus*, *N. humator* and *N. vespillo*. Previous studies found *O. thoracicum* and *T. rugosus* to be associated with spring (Esh & Oxbrough, 2021; Kočárek, 2003; Matuszewski et al., 2010; Růžička, 1994). Matuszewski et al. (2010) reported *O. thoracicum* exclusively during spring. In addition, the other Silphinae species we observed with higher abundances in spring, and that have been documented on carrion in spring, were *N. humator* (Esh & Oxbrough, 2021; Růžička, 1994), *N. vespillo* (Dekeirsschieter et al., 2011; Kočárek, 2003) and *T. sinuatus* (Růžička, 1994). For *N. littoralis*, no such seasonal preference is known (Matuszewski et al., 2010). Instead, *N. littoralis* has been reported to colonize carrion with minimal or absent colonization by blow flies (Calliphoridae). In a study using 90 pig carcasses, the majority (56 carcasses) was monopolized by blow fly larvae and only two by *N. littoralis* with the highest colonization scores for this beetle species in early spring (Matuszewski & Mądra‐Bielewicz, 2021). In our study, we captured a total Diptera larvae volume (mainly made up of blow flies) of only 519 mL in spring, compared to 3208 mL we captured in summer (Appendix 25). Thus, in line with previous findings (Matuszewski & Mądra‐Bielewicz, 2021), the seasonal changes in *N. littoralis* abundance may be explained by resource competition with dipteran larvae. This resource competition could also account for the higher total Silphinae abundance detected during spring when Diptera abundance was lower.

#### 4.3.3 Temperature

Temperature had a positive effect on Silphinae abundance, supporting numerous studies showing a positive relationship of temperature and arthropod diversity and abundance (Baz et al., 2007; Chen et al., 2009; De Jong & Chadwick, 1999; Farwig et al., 2014; Martin‐Piera & Lobo, 1993; Von Hoermann et al., 2018). However, in our study, the abundances of only two of the five species were affected by temperature and the effects were not consistent throughout the seasons. The abundance of both species increased with temperature during spring. In contrast, in summer *O. thoracicum* abundance did not respond to temperature and the abundance of *T. rugosus* decreased with increasing temperature. These changes of effect are likely related to temperature differences between the seasons. During spring, the average temperature was at 8°C. With known lower temperature activity thresholds of 12.0°C for *T. rugosus* (Matuszewski & Szafałowicz, 2013), the temperature may have been too low for activity. As temperatures increase the threshold of thermal inactivity may be passed, resulting in a stronger effect of temperature on Silphinae abundance, like that of summer (average temperature = 15°C). However, information on the thermal ecology of Silphinae, particularly *Nicrophorus* of the tribe Nicrophorini (Merrick & Smith, 2004) is sparse, and in general, there is little known about the biology and ecology of the tribe Silphini (Ikeda et al., 2007; Ratcliffe, 1996).

## 5 CONCLUSIONS

Our experimental carrion study on one of the major subfamilies of beetles involved in carrion decomposition, the Silphinae (Staphylinidae), provided new insights into ecological drivers of their diversity and abundance. Contrary to our assumptions, carrion body mass neither had a distinct nor consistent effect on Silphinae diversity. Our expectations for higher Silphinae abundance and species richness at larger carrion were partially met. Most prominently, our results highlighted carrion decomposition as an important driver of Silphinae diversity. Peaks of Silphinae abundance and species richness on the days with the highest total number of carrion decomposition stages indicate species‐specific preferences for carrion decomposition stages. The abiotic factors temperature, elevation and season affected the Silphinae diversity as already observed for insect communities. To identify these patterns, we used transformation models. With transformation models, there is no need to decide on fixed distributions, they perform very well for data with complex distributions that would hamper classical models with a priori selected types of families. As this data distribution situation is rather common in ecological studies, we expect an increased use of transformation models in ecological research.

## AUTHOR CONTRIBUTIONS

**Gwen Büchner:** Data curation (equal); formal analysis (equal); investigation (equal); visualization (equal); writing – original draft (lead); writing – review and editing (equal). **Torsten Hothorn:** Data curation (equal); formal analysis (lead); software (lead); writing – review and editing (equal). **Heike Feldhaar:** Supervision (supporting); writing – review and editing (equal). **Christian von Hoermann:** Investigation (equal); writing – review and editing (equal). **Tomáš Lackner:** Investigation (supporting); writing – review and editing (equal). **Janine Rietz:** Investigation (equal); writing – review and editing (equal). **Jens Schlüter:** Investigation (equal); writing – review and editing (equal). **Oliver Mitesser:** Writing – review and editing (equal). **M. Eric Benbow:** Writing – review and editing (equal). **Marco Heurich:** Methodology (equal); supervision (equal); writing – review and editing (equal). **Jörg Müller:** Conceptualization (lead); formal analysis (equal); methodology (equal); supervision (equal); writing – original draft (supporting); writing – review and editing (equal).

## CONFLICT OF INTEREST STATEMENT

The authors declare that they have no deliberate competing financial interests or personal relationships that could have influenced the work presented in this paper.

## Data Availability

The data (and the annotated R code, to enable reproduction of the statistical analyses and figures) that support the findings of this study are openly available in Dryad at https://doi.org/10.5061/dryad.xd2547drq.

## APPENDIX 1 Elevation of sites with maximum and minimum elevation for each site in meters above sea level (a.s.l.).

**Table:**

Site	Minimum–Maximum elevation [m]
1	809–817
2	734–768
3	1147–1266
4	1199–1286
5	1167–1222

## APPENDIX 2 Presence of the carrion throughout the experiment.

**Table:**

Carrion species	Site	Spring				Summer			
		Day 4	Day 8	Day 16	Day 23	Day 4	Day 8	Day 16	Day 23
Stoat	1								
	2								
	3								
	4								
	5								
Rat	1								
	2								
	3								
	4								
	5								
Marten	1								
	2								
	3								
	4								
	5								
Raccoon	1								
	2								
	3								
	4								
	5								
Red fox	1								
	2								
	3								
	4								
	5								
Badger	1								
	2								
	3								
	4								
	5								
Beaver	1								
	2								
	3								
	4								
	5								
Roe deer	1								
	2								
	3								
	4								
	5								
Wild boar	1								
	2								
	3								
	4								
	5								
Red deer	1								
	2								
	3								
	4								
	5								

*Note*: Carrion replicas (given by the sites) are listed per carrion species and per season. Grey cells indicate carrion presence, and white cells indicate absence of the carrion due to removal by vertebrate or invertebrate scavengers.

## APPENDIX 4 Formulas of the used models.

**Table:**

Model name		Model formula
EM1	←	Silphinae abundance ~ season * [day + T + log_10_ (carrion body mass) + elevation] + (1|$\frac{\text{site}}{\mathrm{ID}\;\text{carrion}}$)
EM2	←	Silphinae species richness ~ season * [day + T + log_10_ (carrion body mass) + log_10_ (Silphinae abundance) + elevation] + (1|$\frac{\text{site}}{\mathrm{ID}\;\text{carrion}}$)

*Note*: Day stands for day since exposure of carrion, T refers to temperature and ID carrion stands for the individual carcasses (with a unique identifier).

## APPENDIX 6 Effects of the predictors on Silphinae abundance, species richness and the abundances of the five most common Silphinae species given as estimated log‐odds ratios with standard error (model used: Silphinae abundance: M1; Silphinae species richness: M3; for models see Table 2).

**Table:**

Predictors	Estimate ± SE						
	Ab.	Rich.	*T. sin*.	*N. lit*.	*O. tho*.	*T. rug*.	*N. ves*.
Summer	**−3.99 ± 1.8***	−3.07 ± 2.16	−0.17 ± 2.15	−14.99 ± 4.80 × 10^2^	−0.24 ± 2.25	**−4.51 ± 2.06***	−11.13 ± 57.79
Day 8	0.33 ± 0.55	**−1.40 ± 0.75˙**	−0.58 ± 0.72	−13.18 ± 4.80 × 10^2^	0.58 ± 0.58	0.12 ± 0.87	−8.76 ± 57.76
Day 16	**−1.47 ± 0.46****	**−3.10 ± 0.69*****	**−1.53 ± 0.65***	−13.68 ± 4.80 × 10^2^	−0.24 ± 0.50	**−2.05 ± 0.69****	−9.75 ± 57.76
Day 23	**−4.72 ± 0.67*****	**−1.61 ± 0.81***	**−5.16 ± 0.78*****	−18.68 ± 4.80 × 10^2^	**−1.69 ± 0.71***	**−3.73 ± 0.81*****	−11.23 ± 57.76
Temperature	**−0.13 ± 0.07˙**	−0.05 ± 0.07	−0.03 ± 0.07	0.01 ± 0.07	**−0.27 ± 0.09****	**−0.23 ± 0.07****	−0.09 ± 0.07
Abundance of ind.	–	**−6.95 ± 0.56*****	–	–	–	–	–
Carrion body mass	**−0.94 ± 0.24*****	−0.20 ± 0.24	**−0.71 ± 0.26****	**−1.91 ± 0.40*****	**−0.85 ± 0.25*****	**−0.84 ± 0.26****	0.22 ± 0.27
Su. Day 8	−0.55 ± 0.68	1.31 ± 0.89	−0.51 ± 0.85	11.61 ± 4.80 × 10^2^	−0.55 ± 0.73	−0.09 ± 0.99	10.51 ± 57.77
Su. Day 16	**2.29 ± 0.59*****	**4.41 ± 0.85*****	0.88 ± 0.78	12.74 ± 4.80 × 10^2^	**1.55 ± 0.69***	**2.91 ± 0.84*****	11.9 ± 57.76
Su. Day 23	**6.93 ± 0.84*****	**3.28 ± 1.03****	**7.19 ± 1.01*****	18.37 ± 4.80 × 10^2^	19.35 ± 7.67 × 10^2^	**6.35 ± 1.17*****	14.11 ± 57.77
Su. Temperature	0.14 ± 0.14	0.15 × 10^−2^ ± 0.16	−0.15 ± 0.15	−0.07 ± 0.17	0.1 ± 0.17	**0.27 ± 0.15˙**	−0.07 ± 0.15
Su. Abundance of ind.	–	**−0.86 ± 0.47˙**	–	–	–	–	–
Su. Carrion body mass	0.42 ± 0.29	−0.16 ± 0.33	0.1 ± 0.34	**0.82 ± 0.47˙**	0.16 ± 0.35	0.34 ± 0.35	0.08 ± 0.33

*Note*: Statistically significant and marginally significant effects are in bold print and the significance is coded (statistically significant: *p* < .001 = ‘***’, *p* < .01 = ‘**’, *p* < .05 = ‘*’; statistically marginally significant: *p* < .1 = ‘**˙**’). Negative estimates indicate positive biological effects.

Abbreviations: Ab., Silphinae abundance; Abundance of ind., abundance of individuals; *N. lit*., *Necrodes littoralis*; *N. ves*., *Nicrophorus vespilloides*; *O. tho*., *Oiceoptoma thoracicum*; Rich., Silphinae species richness; Su., Summer; *T. rug*., *Thanatophilus rugosus*; *T. sin*., *Thanatophilus sinuatus*.

## APPENDIX 7 Results for the effects of the predictors on Silphinae abundance and species richness with estimates, standard errors, *z*‐values, exponents and *p*‐values.

**Table:**

Silphinae abundance with consideration of carrion body mass				
Predictors	Estimate	SE	*z*‐value	*p*‐value
Summer	**−3.99**	**1.80**	**−2.22**	**.027**
Day 8	0.334	0.551	0.605	.545
Day 16	**−1.47**	**0.464**	**−3.16**	**1.58 × 10** ^**−3**^
Day 23	**−4.72**	**0.672**	**−7.02**	**2.25 × 10** ^**−12**^
Temperature	−0.130	0.0731	−1.77	.0762
Carrion mass	**−0.942**	**0.235**	**−4.01**	**6.13 × 10** ^**−5**^
Su. Day 8	−0.550	0.679	−0.810	.418
Su. Day 16	**2.29**	**0.594**	**3.86**	**1.13 × 10** ^**−4**^
Su. Day 23	**6.93**	**0.835**	**8.29**	**2.20 × 10** ^**−16**^
Su. Temperature	0.142	0.135	1.05	.293
Su. Carrion mass	0.420	0.294	1.43	.153

Silphinae abundance with consideration of carrion species identity				
Predictors	Estimate	SE	*z*‐value	*p*‐value
Summer	−2.99	1.82	−1.65	9.99 × 10^−2^
Day 8	0.265	0.546	0.486	.627
Day 16	**−1.52**	**0.465**	**−3.26**	**1.13 × 10** ^**−3**^
Day 23	**−4.82**	**0.673**	**−7.16**	**7.92 × 10** ^**−13**^
Temperature	−0.132	0.0701	−1.88	.0597
*Mustela erminea/nivalis*	**2.87**	**0.851**	**3.37**	**7.41 × 10** ^**−4**^
*Rattus norvegicus*	**3.24**	**0.886**	**3.66**	**2.53 × 10** ^**−4**^
*Martes martes/foina*	0.524	0.763	0.687	.492
*Procyon lotor*	**1.67**	**0.828**	**2.02**	**.0436**
*Vulpes vulpes*	1.29	0.736	1.75	.0793
*Meles meles*	0.568	0.708	0.803	.422
*Castor fiber*	0.918	0.733	1.25	.211
*Capreolus capreolus*	**1.61**	**0.775**	**2.07**	**.0381**
*Cervus elaphus*	0.584	0.712	0.821	.412
Su. Day 8	−0.477	0.673	−0.709	.478
Su. Day 16	**2.36**	**0.594**	**3.97**	**7.06 × 10** ^**−5**^
Su. Day 23	**7.01**	**0.838**	**8.36**	**2.20 × 10** ^**−16**^
Su. Temperature	0.149	0.133	1.12	.262
Su. *Mustela erminea/nivalis*	−1.95	1.10	−1.78	.0753
Su. *Rattus norvegicus*	−1.60	1.12	−1.43	.152
Su. *Martes martes/foina*	0.641	1.03	0.619	.536
Su. *Procyon lotor*	−1.69	1.08	−1.56	.118
Su. *Vulpes vulpes*	−0.332	1.01	−0.326	.744
Su. *Meles meles*	−0.204	0.974	−0.209	.834
Su. *Castor fiber*	−0.697	0.987	−0.706	.480
Su. *Capreolus capreolus*	−1.13	1.02	−1.11	.267
Su. *Cervus elaphus*	−0.642	0.969	−0.662	.508

Silphinae species richness with consideration of carrion body mass				
Predictors	Estimate	SE	*z*‐value	*p*‐value
Summer	−3.07	2.16	−1.42	.16
Day 8	−1.40	0.746	−1.87	.0612
Day 16	**−3.10**	**0.690**	**−4.49**	**7.28 × 10** ^**−6**^
Day 23	**−1.61**	**0.812**	**−1.98**	**.0477**
Temperature	−0.0511	0.0660	−0.774	.439
Carrion mass	−0.204	0.240	−0.851	.395
Abundance individuals	**−6.95**	**0.563**	**−12.3**	**2.20 × 10** ^**−16**^
Su. Day 8	1.31	0.887	1.48	.138
Su. Day 16	**4.41**	**0.852**	**5.18**	**2.27 × 10** ^**−7**^
Su. Day 23	**3.28**	**1.03**	**3.19**	**1.43 × 10** ^**−3**^
Su. Temperature	−1.47 × 10 ^−3^	0.158	−9.30 × 10 ^−3^	.993
Su. Carrion mass	−0.160	0.328	−0.487	.626
Su. Abundance individuals	−0.857	0.474	−1.81	.0707

Silphinae species richness with consideration of carrion species identity				
Predictors	Estimate	SE	*z*‐value	*p*‐value
Summer	−2.56	2.21	−1.16	.25
Day 8	**−1.69**	**0.775**	**−2.18**	**.0291**
Day 16	**−3.43**	**0.714**	**−4.81**	**1.53 × 10** ^**−6**^
Day 23	**−1.90**	**0.831**	**−2.29**	**.0222**
Temperature	−0.0343	0.0661	−0.519	.604
Abundance individuals	**−7.25**	**0.545**	**−13.3**	**2.20 × 10** ^**−16**^
*Mustela erminea/nivalis*	0.651	0.876	0.743	.457
*Rattus norvegicus*	1.20	0.910	1.32	.188
*Martes martes/foina*	0.880	0.778	1.13	.258
*Procyon lotor*	−0.0938	0.752	−0.125	.901
*Vulpes vulpes*	1.14	0.846	1.35	.178
*Meles meles*	**1.64**	**0.771**	**2.13**	**.0331**
*Castor fiber*	1.39	0.759	1.84	.0660
*Capreolus capreolus*	0.586	0.777	0.754	.451
*Cervus elaphus*	0.448	0.731	0.613	.540
Su. Day 8	1.58	0.907	1.74	.0810
Su. Day 16	**4.83**	**0.869**	**5.56**	**2.66 × 10** ^**−8**^
Su. Day 23	**3.60**	**1.05**	**3.45**	**5.71 × 10** ^**−4**^
Su. Temperature	−0.0109	0.158	−0.0689	.945
Su. Abundance individuals	−0.771	0.479	−1.61	.108
Su. *Mustela erminea/nivalis*	0.489	1.21	0.404	.686
Su. *Rattus norvegicus*	−0.585	1.24	−0.470	.638
Su. *Martes martes/foina*	−0.873	1.05	−0.832	.406
Su. *Procyon lotor*	−0.805	1.00	−0.805	.421
Su. *Vulpes vulpes*	−1.23	1.10	−1.12	.263
Su. *Meles meles*	**−3.20**	**1.02**	**−3.13**	**1.76 × 10** ^**−3**^
Su. *Castor fiber*	−0.985	1.02	−0.971	.332
Su. *Capreolus capreolus*	−0.851	1.05	−0.810	.418
Su. *Cervus elaphus*	−1.19	1.02	−1.17	.241

*Note*: Results of the models with consideration of carrion body mass and with consideration of carrion species identity, respectively, are shown. Reference for carrion species was *Sus scrofa*, reference for sampling day was day 4, and reference season was spring. Significant *p*‐values (*p* < .05) are bold and black, marginally significant *p*‐values (.05 < *p* < .10) are black and non‐significant *p*‐values (*p* ≥ .10) grey. Su., summer.

## APPENDIX 8 Effects of the predictors on Silphinae abundance, species richness and the abundances of the five most common Silphinae species five most common Silphinae species given as estimated log‐odds ratios with standard error (model used: Silphinae abundance: M2; Silphinae species richness: M4; for models see Table 2).

**Table:**

Predictors	Estimate ± SE						
	Ab.	Rich.	*T. sin*.	*N. lit*.	*O. tho*.	*T. rug*.	*N. ves*.
Summer	**−2.99 ± 1.82˙**	−2.56 ± 2.21	0.49 × 10^−2^ ± 2.19	−7.45 ± 41.03	0.45 ± 2.31	−2.7 ± 2.33	−17.75 ± 19.26 × 10^2^
Day 8	0.27 ± 0.55	**−1.69 ± 0.78***	−0.64 ± 0.73	−8.27 ± 40.97	0.52 ± 0.57	0.09 ± 0.87	−16.47 ± 19.26 × 10^2^
Day 16	**−1.52 ± 0.47****	**−3.43 ± 0.71*****	**−1.6 ± 0.66***	−8.74 ± 40.97	−0.27 ± 0.50	**−2.01 ± 0.69****	−17.49 ± 19.26 × 10^2^
Day 23	**−4.82 ± 0.67*****	**−1.90 ± 0.83***	**−5.28 ± 0.79*****	−13.98 ± 40.97	**−1.77 ± 0.71***	**−3.82 ± 0.86*****	−19.35 ± 19.26 × 10^2^
Temperature	**−0.13 ± 0.07˙**	−0.03 ± 0.07	−0.03 ± 0.07	0.04 ± 0.08	**−0.26 ± 0.09****	**−0.23 ± 0.09****	−0.07 ± 0.07
Abundance of ind.	–	**−7.25 ± 0.55*****	–	–	–	–	–
*M. erminea/nivalis*	**2.87 ± 0.85*****	0.65 ± 0.88	**2.34 ± 1.05***	18.43 ± 7.03 × 10^2^	**2.15 ± 0.92***	**3.16 ± 0.99****	−0.17 ± 0.89
*R. norvegicus*	**3.24 ± 0.89*****	1.20 ± 0.91	**2.27 ± 1.05***	18.98 ± 8.76 × 10^2^	**2.86 ± 0.96****	**2.35 ± 0.89****	**2.39 ± 1.29˙**
*M. martes/foina*	0.52 ± 0.76	0.88 ± 0.78	−0.31 ± 0.88	**2.14 ± 1.06***	0.19 ± 0.83	0.63 ± 0.79	0.1 ± 0.93
*P. lotor*	**1.67 ± 0.83**	−0.09 ± 0.75	0.8 ± 0.94	**2.37 ± 1.20***	1.34 ± 0.88	1.39 ± 0.86	0.94 ± 0.97
*V. vulpes*	**1.29 ± 0.74˙**	1.14 ± 0.85	0.25 ± 0.85	**1.92 ± 0.99˙**	0.87 ± 0.79	**1.54 ± 0.83˙**	1.63 ± 1.07
*M. meles*	0.57 ± 0.71	**1.64 ± 0.77***	−0.43 ± 0.83	0.99 ± 0.91	0.52 ± 0.79	0.5 ± 0.74	0.8 ± 0.96
*C. fiber*	0.92 ± 0.73	**1.39 ± 0.76˙**	0.69 ± 0.86	**2.96 ± 1.10****	0.31 ± 0.78	**1.53 ± 0.82˙**	**2.43 ± 1.28˙**
*C. capreolus*	**1.61 ± 0.78***	0.59 ± 0.78	0.91 ± 0.89	1.04 ± 0.95	1.07 ± 0.85	**2.2 ± 0.90***	1.11 ± 1.02
*C. elaphus*	0.58 ± 0.71	0.45 ± 0.73	1.76 × 10^−4^ ± 0.84	0.81 ± 0.88	0.19 ± 0.78	0.19 ± 0.70	**2.5 ± 1.29˙**
Su. Day 8	−0.48 ± 0.67	**1.58 ± 0.91˙**	−0.44 ± 0.85	6.67 ± 40.97	−0.53 ± 0.72	−0.04 ± 1.00	18.21 ± 19.26 × 10^2^
Su. Day 16	**2.36 ± 0.59*****	**4.83 ± 0.87*****	0.99 ± 0.78	7.73 ± 40.97	**1.59 ± 0.69***	**2.85 ± 0.84*****	19.73 ± 19.26 × 10^2^
Su. Day 23	**7.01 ± 0.84*****	**3.60 ± 1.05*****	**7.33 ± 1.03*****	13.74 ± 40.98	36.03 ± 1.00 × 10^4^	**6.44 ± 1.24*****	22.28 ± 19.26 × 10^2^
Su. Temperature	0.15 ± 0.13	−0.01 ± 0.16	−0.15 ± 0.15	−0.13 ± 0.17	0.11 ± 0.17	0.23 ± 0.18	−0.07 ± 0.15
Su. Abundance of ind.	–	−0.77 ± 0.48	–	–	–	–	–
Su. *M. erminea/nivalis*	**−1.95 ± 1.10˙**	0.49 ± 1.21	−1.52 ± 1.31	−16.65 ± 7.03 × 10^2^	−1.5 ± 1.29	**−3.05 ± 1.29***	−0.59 ± 1.16
Su. *R. norvegicus*	−1.6 ± 1.12	−0.58 ± 1.24	0.36 ± 1.38	−0.97 ± 19.87 × 10^2^	−1.02 ± 1.42	−0.34 ± 1.48	**−3.28 ± 1.48***
Su. *M. martes/foina*	0.64 ± 1.03	−0.87 ± 1.05	0.98 ± 1.16	15.01 ± 11.43 × 10^2^	0.48 ± 1.25	−0.28 ± 1.14	0.14 ± 1.22
Su. *P. lotor*	−1.69 ± 1.08	−0.80 ± 1.00	−1.29 ± 1.2	**−2.39 ± 1.35˙**	−0.68 ± 1.27	−1.25 ± 1.17	−1.91 ± 1.24
Su. *V. vulpes*	−0.33 ± 1.02	−1.23 ± 1.10	0.21 ± 1.14	−0.41 ± 1.26	−0.21 ± 1.21	−1.32 ± 1.17	**−2.37 ± 1.30˙**
Su. *M. meles*	−0.2 ± 0.97	**−3.20 ± 1.02****	0.62 ± 1.11	0.3 ± 1.17	−1.09 ± 1.15	−0.68 ± 1.07	**−2.37 ± 1.19***
Su. *C. fiber*	−0.7 ± 0.99	−0.99 ± 1.02	−0.56 ± 1.14	−1.92 ± 1.30	−0.47 ± 1.16	−1.4 ± 1.14	**−3.14 ± 1.47***
Su. *C. capreolus*	−1.13 ± 1.02	−0.85 ± 1.05	−0.41 ± 1.17	−0.34 ± 1.17	−1.62 ± 1.20	−1.79 ± 1.22	−0.92 ± 1.28
Su. *C. elaphus*	−0.64 ± 0.97	−1.19 ± 1.02	0.05 ± 1.11	−0.97 ± 1.07	−1.32 ± 1.13	−0.98 ± 1.00	−1.56 ± 1.55

*Note*: Statistically significant and marginally significant effects are in bold print and the significance is coded (statistically significant: *p* < .001 = ‘***’, *p* < .01 = ‘**’, *p* < .05 = ‘*’; statistically marginally significant: *p* < .1 = ‘**˙**’). Note that negative estimates indicate positive effects in transformation models.

Abbreviations: Ab., Silphinae abundance; Abundance of ind., abundance of individuals; *C. capreolus*, *Capreolus capreolus*; *C. elaphus*, *Cervus elaphus*; *C. fiber*, *Castor fiber*; *M. erminea/nivalis*, *Mustela erminea/nivalis*; *M. martes/foina*, *Martes martes/foina*; *M. meles*, *Meles meles*; *N. lit*., *Necrodes littoralis*; *N. ves*., *Nicrophorus vespilloides*; *O. tho*., *Oiceoptoma thoracicum*; *P. lotor*, *Procyon lotor*; *R. norvegicus*, *Rattus norvegicus*; Rich., Silphinae species richness; Su., Summer; *T. rug*., *Thanatophilus rugosus*; *T. sin*., *Thanatophilus sinuatus*; *V. vulpes*, *Vulpes vulpes*.

## APPENDIX 9 Results for the effects of the predictors on abundance of the five most common Silphinae species with estimates, standard errors, *z*‐values, exponents and *p*‐values.

**Table:**

*Oiceoptoma thoracicum*				
With consideration of carrion species identity				
Fixed effect	Estimate	SE	*z*‐value	*p*‐value
Summer	0.451538	2.308040	0.1956	.844894
*Mustela erminea/nivalis*	2.150328	0.924126	2.3269	**.019972**
*Rattus norvegicus*	2.857924	0.957027	2.9863	**.002824**
*Martes martes/foina*	0.193187	0.828159	0.2333	.815550
*Procyon lotor*	1.336437	0.880649	1.5176	.129126
*Vulpes vulpes*	0.873056	0.787145	1.1091	.267369
*Meles meles*	0.518490	0.786093	0.6596	.509524
*Castor fiber*	0.309428	0.783469	0.3949	.692882
*Capreolus capreolus*	1.070628	0.854683	1.2527	.210329
*Cervus elaphus*	0.190885	0.779025	0.2450	.806433
Day 8	0.521350	0.567943	0.9180	.358639
Day 16	−0.272133	0.500194	−0.5441	.586404
Day 23	−1.770977	0.710908	−2.4911	**.012733**
Temperature	−0.256484	0.091002	−2.8184	**.004826**
Su. *Mustela erminea/nivalis*	−1.504830	1.289067	−1.1674	.243058
Su. *Rattus norvegicus*	−1.022521	1.423338	−0.7184	.472513
Su. *Martes martes/foina*	0.478471	1.245676	0.3841	.700900
Su. *Procyon lotor*	−0.676539	1.271774	−0.5320	.594750
Su. *Vulpes vulpes*	−0.212977	1.212985	−0.1756	.860623
Su. *Meles meles*	−1.090069	1.150067	−0.9478	.343216
Su. *Castor fiber*	−0.471016	1.159576	−0.4062	.684598
Su. *Capreolus capreolus*	−1.622228	1.202708	−1.3488	.177397
Su. *Cervus elaphus*	−1.317155	1.131980	−1.1636	.244593
Su. Day 8	−0.531073	0.720927	−0.7367	.461333
Su. Day 16	1.585618	0.688475	2.3031	**.021274**
Su. Day 23	36.032046	9999.949005	0.0036	.997125
Su. Temperature	0.112377	0.165598	0.6786	.497382

With consideration of carrion body mass				
Fixed effect	Estimate	SE	*z*‐value	*p*‐value
Summer	−0.244511	2.248634	−0.1087	.913410
Day 8	0.582769	0.575735	1.0122	.311434
Day 16	−0.241569	0.501660	−0.4815	.630134
Day 23	−1.688218	0.710286	−2.3768	**.017463**
Temperature	−0.266353	0.093575	−2.8464	**.004421**
Carrion mass	−0.850894	0.252029	−3.3762	**.000735**
Su. Day 8	−0.553235	0.726069	−0.7620	.446084
Su. Day 16	1.553000	0.687991	2.2573	**.023989**
Su. Day 23	19.348466	767.385338	0.0252	.979885
Su. Temperature	0.100801	0.165863	0.6077	.543360
Su. Carrion mass	0.164364	0.350255	0.4693	.638877

*Necrodes littoralis*				
With consideration of carrion species identity				
Fixed effect	Estimate	SE	*z*‐value	*p*‐value
Summer	−7.449123	41.032322	−0.1815	.855942
*Mustela erminea/nivalis*	18.430032	702.876169	0.0262	.979081
*Rattus norvegicus*	18.983808	875.720333	0.0217	.982705
*Martes martes/foina*	2.144478	1.061779	2.0197	**.043414**
*Procyon lotor*	2.370569	1.198800	1.9775	**.047991**
*Vulpes vulpes*	1.919639	0.986209	1.9465	.051597
*Meles meles*	0.994333	0.908464	1.0945	.273726
*Castor fiber*	2.962537	1.097583	2.6991	**.006952**
*Capreolus capreolus*	1.036347	0.952249	1.0883	.276456
*Cervus elaphus*	0.812518	0.875057	0.9285	.353132
Day 8	−8.272264	40.970378	−0.2019	.839988
Day 16	−8.740730	40.968294	−0.2134	.831051
Day 23	−13.982864	40.969980	−0.3413	.732881
Temperature	0.036192	0.076265	0.4746	.635104
Su. *Mustela erminea/nivalis*	−16.647950	702.876611	−0.0237	.981104
Su. *Rattus norvegicus*	−0.965174	1987.154495	−0.0005	.999612
Su. *Martes martes/foina*	15.014818	1143.007048	0.0131	.989519
Su. *Procyon lotor*	−2.394374	1.349973	−1.7736	.076122
Su. *Vulpes vulpes*	−0.406992	1.264995	−0.3217	.747654
Su. *Meles meles*	0.300536	1.173765	0.2560	.797917
Su. *Castor fiber*	−1.920332	1.299506	−1.4777	.139477
Su. *Capreolus capreolus*	−0.344915	1.167367	−0.2955	.767639
Su. *Cervus elaphus*	−0.973165	1.073464	−0.9066	.364637
Su. Day 8	6.667187	40.974218	0.1627	.870742
Su. Day 16	7.732323	40.972274	0.1887	.850312
Su. Day 23	13.740373	40.975428	0.3353	.737375
Su. Temperature	−0.129337	0.174504	−0.7412	.458591

With consideration of carrion body mass				
Fixed effect	Estimate	SE	*z*‐value	*p*‐value
Summer	−14.989815	479.571484	−0.0313	.97506
Day 8	−13.184408	479.567182	−0.0275	.97807
Day 16	−13.675729	479.566949	−0.0285	.97725
Day 23	−18.675167	479.566683	−0.0389	.96894
Temperature	0.010388	0.074095	0.1402	.88850
Carrion mass	−1.911703	0.397520	−4.8091	**1.516e‐06**
Su. Day 8	11.614904	479.567739	0.0242	.98068
Su. Day 16	12.735858	479.567376	0.0266	.97881
Su. Day 23	18.372727	479.567223	0.0383	.96944
Su. Temperature	−0.067847	0.168832	−0.4019	.68779
Su. Carrion mass	0.820441	0.465040	1.7642	.07769

*Thanatophilus sinuatus*				
With consideration of carrion species identity				
Fixed effect	Estimate	SE	*z*‐value	*p*‐value
Summer	0.0048983	2.1914509	0.0022	0.99822
*Mustela erminea/nivalis*	2.3373579	1.0544235	2.2167	**0.02664**
*Rattus norvegicus*	2.2712253	1.0498899	2.1633	**0.03052**
*Martes martes/foina*	−0.3100573	0.8810947	−0.3519	0.72491
*Procyon lotor*	0.7958739	0.9384721	0.8481	0.39641
*Vulpes vulpes*	0.2461621	0.8458640	0.2910	0.77104
*Meles meles*	−0.4251351	0.8288473	−0.5129	0.60800
*Castor fiber*	0.6912165	0.8637257	0.8003	0.42355
*Capreolus capreolus*	0.9078772	0.8949162	1.0145	0.31035
*Cervus elaphus*	0.0001758	0.8406707	0.0002	0.99983
Day 8	−0.6386884	0.7281270	−0.8772	0.38040
Day 16	−1.5978957	0.6587481	−2.4257	**0.01528**
Day 23	−5.2794186	0.7884347	−6.6961	**2.141e‐11**
Temperature	−0.0275474	0.0672069	−0.4099	0.68189
Su. *Mustela erminea/nivalis*	−1.5193953	1.3085448	−1.1611	0.24559
Su. *Rattus norvegicus*	0.3614661	1.3845114	0.2611	0.79403
Su. *Martes martes/foina*	0.9764967	1.1605433	0.8414	0.40012
Su. *Procyon lotor*	−1.2940795	1.2042717	−1.0746	0.28257
Su. *Vulpes vulpes*	0.2116544	1.1415147	0.1854	0.85290
Su. *Meles meles*	0.6246065	1.1067071	0.5644	0.57249
Su. *Castor fiber*	−0.5619367	1.1352613	−0.4950	0.62061
Su. *Capreolus capreolus*	−0.4050634	1.1653863	−0.3476	0.72816
Su. *Cervus elaphus*	0.0546427	1.1121649	0.0491	0.96081
Su. Day 8	−0.4411328	0.8467594	−0.5210	0.60239
Su. Day 16	0.9944438	0.7813331	1.2728	0.20311
Su. Day 23	7.3297957	1.0258245	7.1453	**8.982e‐13**
Su. Temperature	−0.1496683	0.1512842	−0.9893	0.32251

With consideration of carrion body mass				
Fixed effect	Estimate	SE	*z*‐value	*p*‐value
Summer	−0.174344	2.145259	−0.0813	.935228
Day 8	−0.580297	0.723645	−0.8019	.422606
Day 16	−1.527070	0.654568	−2.3329	**.019651**
Day 23	−5.159083	0.775531	−6.6523	**2.885e‐11**
Temperature	−0.030463	0.067986	−0.4481	.654098
Carrion mass	−0.713459	0.261921	−2.7239	**.006451**
Su. Day 8	−0.507028	0.845171	−0.5999	.548565
Su. Day 16	0.878544	0.777521	1.1299	.258505
Su. Day 23	7.192691	1.012310	7.1052	**1.201e‐12**
Su. Temperature	−0.148956	0.152028	−0.9798	.327188
Su. Carrion mass	0.100411	0.340483	0.2949	.768064

*Thanatophilus rugosus*				
With consideration of carrion species identity				
Fixed effect	Estimate	SE	*z*‐value	*p*‐value
Summer	−2.698861	2.325670	−1.1605	.2458591
*Mustela erminea/nivalis*	3.162039	0.987057	3.2035	**.0013577**
*Rattus norvegicus*	2.353312	0.889960	2.6443	**.0081863**
*Martes martes/foina*	0.626768	0.791074	0.7923	.4281856
*Procyon lotor*	1.391286	0.861020	1.6159	.1061248
*Vulpes vulpes*	1.539566	0.830590	1.8536	.0637993
*Meles meles*	0.503778	0.744672	0.6765	.4987164
*Castor fiber*	1.529277	0.816668	1.8726	.0611263
*Capreolus capreolus*	2.201530	0.897847	2.4520	**.0142060**
*Cervus elaphus*	0.191826	0.696130	0.2756	.7828858
Day 8	0.088431	0.866617	0.1020	.9187236
Day 16	−2.010175	0.692252	−2.9038	**.0036864**
Day 23	−3.817892	0.860378	−4.4375	**9.103e‐06**
Temperature	−0.228627	0.086847	−2.6325	**.0084757**
Su. *Mustela erminea/nivalis*	−3.047403	1.287924	−2.3661	**.0179748**
Su. *Rattus norvegicus*	−0.341016	1.481738	−0.2301	.8179782
Su. *Martes martes/foina*	−0.280131	1.142494	−0.2452	.8063074
Su. *Procyon lotor*	−1.247781	1.166783	−1.0694	.2848805
Su. *Vulpes vulpes*	−1.323841	1.168880	−1.1326	.2573939
Su. *Meles meles*	−0.677288	1.071858	−0.6319	.5274642
Su. *Castor fiber*	−1.398180	1.138518	−1.2281	.2194204
Su. *Capreolus capreolus*	−1.788932	1.217529	−1.4693	.1417477
Su. *Cervus elaphus*	−0.984239	1.003862	−0.9805	.3268632
Su. Day 8	−0.035237	0.996930	−0.0353	.9718044
Su. Day 16	2.849586	0.840656	3.3897	**.0006996**
Su. Day 23	6.444568	1.240468	5.1953	**2.044e‐07**
Su. Temperature	0.234981	0.179760	1.3072	.1911480

With consideration of carrion body mass				
Fixed effect	Estimate	SE	*z*‐value	*p*‐value
Summer	−4.508929	2.064435	−2.1841	**.0289550**
Day 8	0.120290	0.872486	0.1379	.8903427
Day 16	−2.046375	0.692911	−2.9533	**.0031439**
Day 23	−3.729567	0.807619	−4.6180	**3.875e‐06**
Temperature	−0.233820	0.073217	−3.1935	**.0014055**
Carrion mass	−0.837002	0.255196	−3.2798	**.0010387**
Su. Day 8	−0.094516	0.994751	−0.0950	.9243028
Su. Day 16	2.906523	0.841818	3.4527	**.0005551**
Su. Day 23	6.345276	1.166776	5.4383	**5.379e‐08**
Su. Temperature	0.266555	0.147185	1.8110	.0701378
Su. Carrion mass	0.340959	0.349839	0.9746	.3297504

*Nicrophorus vespilloides*				
With consideration of carrion species identity				
Fixed effect	Estimate	SE	*z*‐value	*p*‐value
Summer	−17.750930	1925.578970	−0.0092	.99264
*Mustela erminea/nivalis*	−0.167506	0.886744	−0.1889	.85017
*Rattus norvegicus*	2.386686	1.292330	1.8468	.06477
*Martes martes/foina*	0.101660	0.929968	0.1093	.91295
*Procyon lotor*	0.935009	0.972172	0.9618	.33616
*Vulpes vulpes*	1.631249	1.069015	1.5259	.12703
*Meles meles*	0.795353	0.963133	0.8258	.40892
*Castor fiber*	2.426166	1.280888	1.8941	.05821
*Capreolus capreolus*	1.106694	1.016918	1.0883	.27647
*Cervus elaphus*	2.503748	1.286107	1.9468	.05156
Day 8	−16.474224	1925.577649	−0.0086	.99317
Day 16	−17.486998	1925.577571	−0.0091	.99275
Day 23	−19.354683	1925.577329	−0.0101	.99198
Temperature	−0.069753	0.073507	−0.9489	.34266
Su. *Mustela erminea/nivalis*	−0.585006	1.155092	−0.5065	.61254
Su. *Rattus norvegicus*	−3.279815	1.483600	−2.2107	**.02706**
Su. *Martes martes/foina*	0.135986	1.218011	0.1116	.91110
Su. *Procyon lotor*	−1.914304	1.235278	−1.5497	.12121
Su. *Vulpes vulpes*	−2.365704	1.300208	−1.8195	.06884
Su. *Meles meles*	−2.374813	1.185032	−2.0040	**.04507**
Su. *Castor fiber*	−3.144830	1.473645	−2.1340	**.03284**
Su. *Capreolus capreolus*	−0.923804	1.280801	−0.7213	.47074
Su. *Cervus elaphus*	−1.555405	1.548570	−1.0044	.31518
Su. Day 8	18.214608	1925.577175	0.0095	.99245
Su. Day 16	19.732339	1925.577098	0.0102	.99182
Su. Day 23	22.280143	1925.576657	0.0116	.99077
Su. Temperature	−0.071895	0.147978	−0.4858	.62708

With consideration of carrion body mass				
Fixed effect	Estimate	SE	*z*‐value	*p*‐value
Summer	−11.129904	57.790305	−0.1926	.8473
Day 8	−8.764874	57.764295	−0.1517	.8794
Day 16	−9.746502	57.761665	−0.1687	.8660
Day 23	−11.227006	57.762397	−0.1944	.8459
Temperature	−0.092754	0.073029	−1.2701	.2040
Carrion mass	0.215360	0.268478	0.8022	.4225
Su. Day 8	10.514903	57.766167	0.1820	.8556
Su. Day 16	11.897244	57.763625	0.2060	.8368
Su. Day 23	14.107648	57.765624	0.2442	.8071
Su. Temperature	−0.074856	0.146198	−0.5120	.6086
Su. Carrion mass	0.075699	0.332353	0.2278	.8198

*Note*: Results of the models are shown per species and for spring and summer deployment respectively. Reference for carrion species was *Sus scrofa*, reference for sampling day was day 4. Significant *p*‐values (*p* < .05) are bold and black, marginally significant *p*‐values (.05 < *p* < .10) are black and non‐significant *p*‐values (*p* ≥ .10) grey.

## APPENDIX 23 Effects of the predictors on Silphinae abundance, species richness and the abundances of the five most common Silphinae species given as estimated log‐odds ratios with standard error (model used: Silphinae abundance + abundance of the five most common Silphinae species individually: EM1; Silphinae species richness: EM2; for models see Appendix 4).

**Table:**

Predictors	Estimate ± SE						
	Ab.	Rich.	*T. sin*.	*N. lit*.	*O. tho*.	*T. rug*.	*N. ves*.
Summer	−1.63 ± 2.84	1.37 ± 3.30	0.69 ± 3.19	−11.6 ± 36.8	5.49 ± 3.58	−6.25 ± 3.87	−18.7 ± 457
Day 8	0.41 ± 0.57	**−1.39 ± 0.76˙**	−0.57 ± 0.74	−7.99 ± 36.6	0.77 ± 0.6	0.05 ± 0.86	−13.0 ± 457
Day 16	**−1.50 ± 0.47****	**−3.21 ± 0.71*****	**−1.57 ± 0.67***	−8.52 ± 36.6	−0.25 ± 0.51	**−2.0 ± 0.69****	−13.9 ± 457
Day 23	**−4.41 ± 0.70*****	**−1.62 ± 0.82***	**−4.94 ± 0.78*****	−13.2 ± 36.6	**−1.29 ± 0.69 ˙**	**−4.13 ± 0.80*****	−15.6 ± 457
Temperature	**−0.19 ± 0.08***	−0.08 ± 0.07	−0.08 ± 0.08	−0.04 ± 0.03	**−0.34 ± 0.09*****	**−0.17 ± 0.08***	−0.06 ± 0.09
Abundance of ind.	–	**−6.93 ± 0.57*****	–	–	–	–	–
Carrion body mass	**−0.95 ± 0.24*****	−0.20 ± 0.24	**−0.71 ± 0.27****	**−1.94 ± 0.4*****	**−0.85 ± 0.25*****	**−0.83 ± 0.25*****	0.22 ± 0.27
Elevation	**4.1** × **10** ^**−3**^ **± 1.2 × 10** ^**−3**^ *******	2.8 × 10^−4^ ±1.4 × 10^−3^	**3.6 × 10** ^**−3**^ **± 1.5 × 10** ^**−3**^*	NaN	**4.5 × 10** ^**−3**^ **± 1.1 × 10** ^**−3**^ *******	**−1.8 × 10** ^**−3**^ **± 4.4 × 10** ^**−4**^ *******	−4.5 × 10^−4^ ± 1.8 × 10^−3^
Su. Day 8	−0.57 ± 0.70	**1.59 ± 0.91˙**	−0.54 ± 0.87	6.29 ± 36.6	−0.56 ± 0.76	−0.05 ± 0.99	14.6 ± 457
Su. Day 16	**2.33 ± 0.60*****	**4.47 ± 0.87*****	0.93 ± 0.79	7.62 ± 36.6	**1.51 ± 0.69***	**2.83 ± 0.83*****	16.1 ± 457
Su. Day 23	**6.69 ± 0.87*****	**3.59 ± 1.05*****	**6.94 ± 1.02*****	12.7 ± 36.6	33.9 ± 1.0 × 10^4^	**6.67 ± 1.13*****	18.3 ± 457
Su. Temperature	0.17 ± 0.15	−0.12 × 10^−2^ ± 0.17	−0.09 ± 0.16	−0.07 ± 0.1	0.07 ± 0.17	**0.21 ± 0.15**	−0.01 ± 0.17
Su. Abundance of ind.	–	**−1.01 ± 0.48***	–	–	–	–	–
Su. Carrion body mass	0.41 ± 0.30	−0.22 ± 0.33	0.1 ± 0.35	**0.88 ± 0.46˙**	0.15 ± 0.35	0.35 ± 0.33	0.1 ± 0.33
Su. Elevation	−2.3 × 10^−3^ ± 1.5 × 10^−3^	−2.5 × 10^−3^ ± 1.6 × 10^−3^	−1.4 × 10^−3^ ± 1.6 × 10^−3^	1.2 × 10^−4^ ± 1.8 × 10^−3^	**−4.6 × 10** ^**−3**^ **± 1.7 × 10** ^**−3**^ ******	−1.9 × 10^−3^ ± 1.9 × 10^−3^	2.1 × 10^−3^ ± 1.8 × 10^−3^

*Note*: Statistically significant and marginally significant effects are in bold print and the significance is coded (statistically significant: *p* < .001 = ‘***’, *p* < .01 = ‘**’, *p* < .05 = ‘*’; statistically marginally significant: *p* < .1 = ‘**˙**’). Negative estimates indicate positive biological effects.

## APPENDIX 24 Silphinae species with total abundances for spring and summer.

**Table:**

Species	Abundance spring	Abundance summer	*p*‐value
*Oiceoptoma thoracicum*	1178	218	**.003**
*Necrodes littoralis*	1252	216	.264
*Thanatophilus rugosus*	1160	126	.230
*Thanatophilus sinuatus*	1669	1248	**.041**
*Nicrophorus vespilloides*	58	186	**<.001**
*Nicrophorus humator*	8	2	.092
*Nicrophorus investigator*	0	20	**<.001**
*Nicrophorus interruptus*	0	7	**.025**
*Nicrophorus vespillo*	4	2	.411*
*Nicrophorus sepultor*	0	2	.157*

*Note*: Wilcoxon rank sum tests were used to detect statistically significant differences of the total abundances between the seasons of deployment. Significant differences are printed bold.

*Sample sizes of *Nicrophorus sepultor* and *Nicrophorus vespillo* were not sufficient for reliable statistical analytics.
